# Optimal wave reflection as a mechanism for seagrass self-organization

**DOI:** 10.1038/s41598-023-46788-4

**Published:** 2023-11-20

**Authors:** Roeland C. van de Vijsel, Emilio Hernández-García, Alejandro Orfila, Damià Gomila

**Affiliations:** 1 IFISC (CSIC-UIB). Institute for Cross-Disciplinary Physics and Complex Systems, 07122 Palma, Mallorca Spain; 2 Now at: Hydrology and Environmental Hydraulics Group, Wageningen University, Wageningen, The Netherlands; 3 IMEDEA (CSIC-UIB). Mediterranean Institute for Advanced Studies, 07190 Esporles, Mallorca Spain

**Keywords:** Physical oceanography, Ecological modelling, Nonlinear phenomena, Emergence, Fluid dynamics

## Abstract

Ecosystems threatened by climate change can boost their resilience by developing spatial patterns. Spatially regular patterns in wave-exposed seagrass meadows are attributed to self-organization, yet underlying mechanisms are not well understood. Here, we show that these patterns could emerge from feedbacks between wave reflection and seagrass-induced bedform growth. We derive a theoretical model for surface waves propagating over a growing seagrass bed. Wave-induced bed shear stress shapes bedforms which, in turn, trigger wave reflection. Numerical simulations show seagrass pattern development once wave forcing exceeds a critical amplitude. In line with Mediterranean Sea field observations, these patterns have half the wavelength of the forcing waves. Our results raise the hypothesis that pattern formation optimizes the potential of seagrass meadows to reflect wave energy, and a clear direction for future field campaigns. If wave-reflecting pattern formation increases ecosystem resilience under globally intensifying wave climates, these ecosystems may inspire nature-based coastal protection measures.

## Introduction

Spatial patterns are ubiquitous in ecosystems around the world^1^, ranging from vegetation patterns in arid ecosystems^2^ and tidal marshes^3,4^, to shellfish reefs^5,6^ and deep-water corals^7^. They can form due to local disturbances^8^ or internal feedbacks^1,9^, and can be spatially irregular or regular, depending on their formation mechanisms^10^. Self-organized ecosystem patterns, i.e. larger-scale structures formed by local feedbacks^1^, are often observed to change their morphology in a systematic way under gradually changing environmental conditions that eventually shift an ecosystem closer to a tipping point^10–12^. This property makes self-organized spatial patterns valuable “resilience indicators” of ecosystem robustness against critical transitions. However, it was recently suggested that spatial patterning, whether self-organized or not, increases ecosystem resilience and can reduce abrupt tipping behavior to a more gradual transition^13^. To understand how spatial patterns affect ecosystem resilience and whether patterning allows ecosystems to mitigate climate change impacts, insight into pattern formation mechanisms is crucial^10,14^.

Seagrass ecosystems exhibit a diversity of spatial patterns, including gaps, spots, hexagons, stripes and rings in wave-dominated environments^15–18^ and stripes and irregular patches in tide-dominated environments^19^. Seagrasses are marine flowering plants that form extensive meadows in intertidal and subtidal coastal seas around the world^20^. Seagrass ecosystems provide biodiverse habitats^16^, improve water quality^21–24^, sequester carbon^25^ and provide coastal protection by preventing erosion^26^ and damping waves^27,28^. Understanding how seagrass patterns affect the resilience of these key marine systems is therefore essential. Seagrass patterns in tidal environments have been explained by interactions between hydrodynamic scour and gradients of light limitation and dessication^19^. Expanding ring structures in wave-exposed meadows have been explained by self-inflicted sulfide posioning^18^. In environments such as the Mediterranean Sea, where tidal influence is generally limited and wave forcing is more important, striped seagrass bands^16^ and hexagonal gap patterns^15,17^ have also been suggested to be formed by self-organization, however the underlying mechanisms have not been clearly identified yet. As climate change is associated with increasing wind energy and ocean wave power^29,30^, we focus our study on these wave-exposed meadows and their spatially regular patterns.

**Figure 1:**
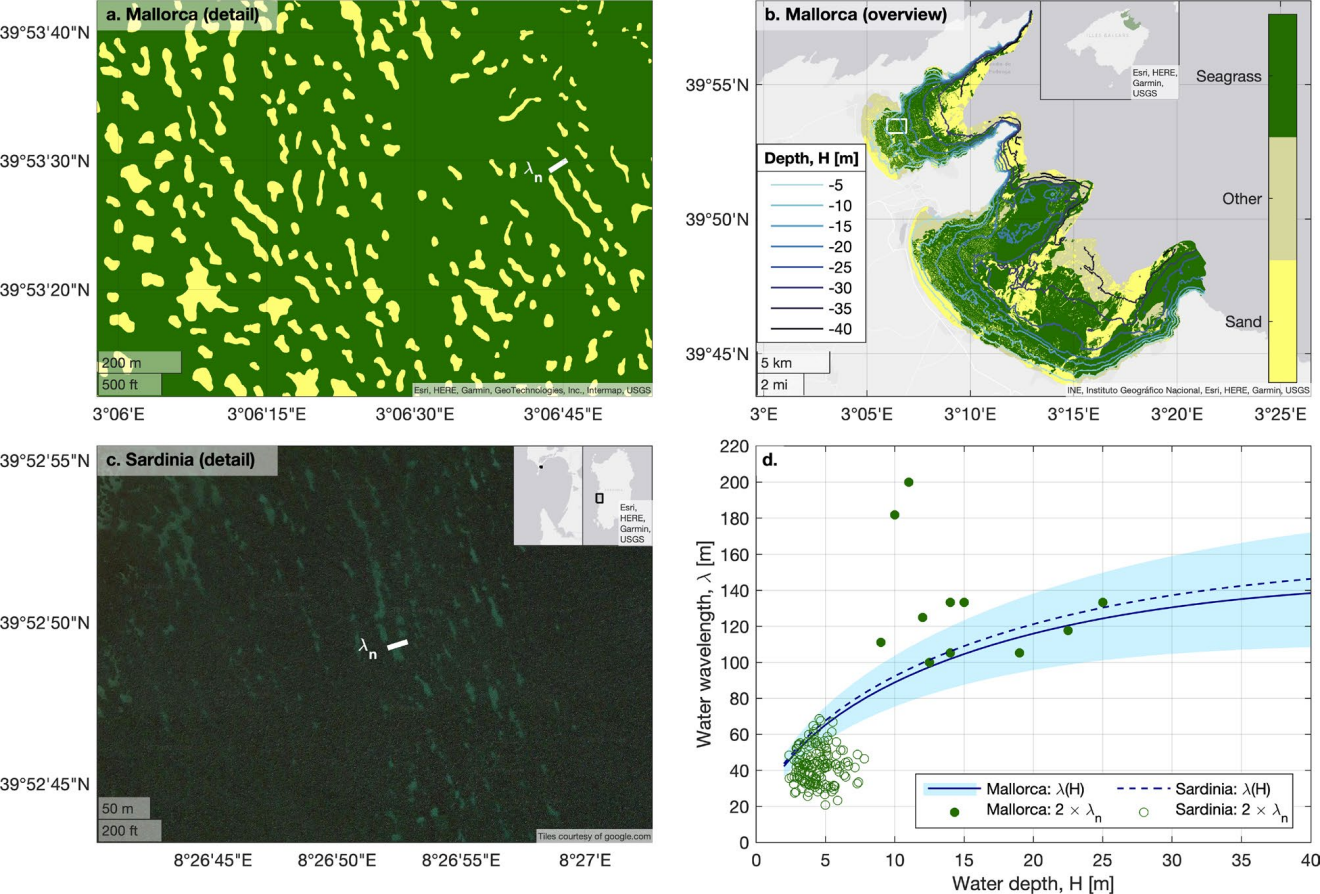
Relation between seagrass patterning, bathymetry and wave conditions. (**a**) Regularly spaced gap patterns in the seagrass meadow at the Bay of Pollença (Mallorca, Spain). The short white line indicates the typical seagrass pattern wavelength (50m) observed around this water depth. Map data: Esri, HERE, Garmin, GeoTechnologies, Inc., Intermap, USGS. (**b**) Overview of the Bays of Pollença and Alcúdia (Mallorca, Spain). Color shadings indicate different substrate types, where “seagrass” refers to *Posidonia oceanica* and “other” refers to other species of seagrass and algae. Blue lines are isobaths. Data of maps a and b derived from the Life Posidonia project^46^. Inset in overview map b shows the area’s location relative to Mallorca. White rectangle indicates where detail map a is located. Main and inset map data: INE, Instituto Geográfico Nacional, Esri, HERE, Garmin, USGS. (**c**) Banded seagrass patterns in the Gulf of Oristano (Sardinia, Italy), visible in satellite imagery. The short white line indicates the typical seagrass pattern wavelength (20m) observed around this water depth. The left inset shows the location of this aerial image within the Gulf of Oristano, while the right inset shows the Gulf of Oristano relative to the island of Sardinia. Main map data ©2023 Imagery ©2023, Airbus, Maxar Technologies. ©2023 Google LLC. Inset map data: Esri, HERE, Garmin, USGS. (**d**) Water wavelengths $\lambda$ as a function of water depth *H*, compared to seagrass pattern wavelengths $\lambda _n$. Based on reanalysed peak wave periods, the 1% events with the highest bed shear-stress are selected, and the wavelengths corresponding to these most energetic waves are obtained. The blue shaded area shows the range between the 2.5% and 97.5% quantiles of the wavelength distribution, for the 1% most energetic waves reanalysed at the Bay of Pollença. The solid blue line indicates the 50% quantile (median). Green filled circles indicate *twice* the observed^15^ seagrass pattern wavelengths (i.e., the typical spacing between patterns) at the Bays of Pollença and Alcúdia. The blue dashed line indicates the water wavelength calculated as a function of depth for the Gulf of Oristano, based on the peak wave period observed offshore^47^. Green open circles indicate *twice* the wavelength of banded seagrass patterns in this gulf^16^. These seagrass wavelength and corresponding water depth data were kindly provided by Coppa et al.^16^ and are here shown with their permission.

To understand the formation of these regular spatial seagrass patterns, we first estimate their spatial dimensions. Banded seagrass patterns (i.e. whose two-dimensional power spectrum has clear directionality^16^) observed at the Gulf of Oristano, Sardinia, Italy (Fig. 1c) are reported to have a typical wavelength (i.e., crest-to-crest length) of around 15–35 m, and are found between 2 and 7 m depth^16^. Gap patterns are found around Mallorca, Spain (Fig. 1a,b). Statistical analysis reveals circular or six-fold symmetry of the two-dimensional power spectrum of the pattern, implying an approximately hexagonal symmetry^15^, so that we will refer to them as “hexagonal gap patterns”. These structures have longer wavelengths, mainly between 50 and 75 m, but are also found at greater depths, roughly from 7 to 25 m^15^. This shortening of pattern wavelengths with shoaling depths suggests a relation with wave forcing, as water surface waves also shorten when they approach shallower waters^31^. Note that within the small domains shown in Fig. 1a,c, pattern geometry might deviate from these domain-averaged pattern characteristics. Both Sardinia and Mallorca are located in the western Mediterranean Sea, a micro-tidal environment where waves are the main hydrodynamic forcing. To have a first quick estimate of the most energetic wave conditions offshore of the Gulf of Oristano and the Bays of Pollença and Alcúdia, we considered openly accessible wave-reanalysis data^32^. Over the period January 2021 and July 2023, peak wave periods $T_p$ 50 km west-southwest off the entrance to the Gulf of Oristano (39.7131$^\circ$N, 7.8786$^\circ$E) and 50 km northeast off the entrance to the Bays of Pollença and Alcúdia (40.2288$^\circ$N, 3.6545$^\circ$E) are distributed with a 99-percentile of 10.7 s and 10.4 s, respectively. Assuming that the wave period does not change while waves travel from deep to shallow waters, the wave dispersion relation can be simplified to express water wavelength $\lambda$ in terms of water depth *H*, i.e. $\lambda = \lambda _\infty \tanh {(2 \pi H / \lambda )}$, where $\lambda _\infty$ is the wavelength in deep waters^31^. Assuming that the two chosen locations are far enough offshore to be considered deep water, their deep-water wavelengths $\lambda _\infty$ can be approximated as $\lambda _\infty \approx g {T_p}^2 / (2 \pi ) = 180$ and 168 m for Sardinia and Mallorca, respectively. This yields $\lambda \approx 47-85$ m for depths between 2 and 7 m at Sardinia and $\lambda \approx 82 - 137$ m for depths between 7 and 25 m at Mallorca. The most energetic waves are thus roughly twice as long as the observed seagrass pattern wavelengths.

This finding points towards Bragg resonance as a possible explanation of these seagrass patterns. Bragg reflection occurs when waves travel across a regularly patterned medium (e.g., light waves travelling across a photonic crystal or water waves propagating over sand ripples). Wave reflection is maximal when the wavelength of the incoming wave is twice the wavelength of the patterned surface, a phenomenon known as Bragg resonance^33^. Bragg reflection has been demonstrated for surface water waves travelling over one-dimensional^33^ as well as two-dimensional^34^ patterned bottoms, through mathematical theory^33–35^, numerical simulation^36^, laboratory^33,34,37^ and field measurements^38^. Interactions between incoming and reflected water waves travelling over static bed undulations can create a partially standing wave pattern, which induces a partially standing bed shear stress pattern that could create new bedforms on erodible beds^39,40^. This has been shown in theory^40,41^, laboratory experiments^39,41–43^ and numerical modelling of laboratory^42^ and field conditions^41^. However, whether this mechanism plays a role in the formation of regularly patterned seagrass meadows has not been investigated so far.

Here, we will test the hypothesis that Bragg reflection can drive the self-organization of seagrass patterns in wave-exposed systems. Although Bragg reflection has been suggested to create bedforms in the absence of biotic processes as well^39–43^, such abiotic bedforms typically have a mostly one-dimensional geometry, i.e. they consist of parallel sand bars or ripples. However, the bedform patterns observed in some seagrass meadows (Fig. 1a,b) have a clear two-dimensional geometry, consisting of hexagonal gaps patterns^15^. As hexagonal patterns have previously been attributed to biological self-organization^15,17,44^ and since hexagonal geometries are not commonly described for abiotic sand bar fields, we expect that biogeomorphic feedbacks play a significant role in the formation of the bedform patterns observed in seagrass meadows (Fig. 1). Our hypothesis is therefore based on a self-reinforcing feedback cycle that involves three biogeomorphic processes. First, wave-induced bed shear stress increases net seagrass mortality^45^. Seagrass growth leads to higher bed elevation, through increased sedimentation and the growth of interwoven rhizomes, leading to the formation and vertical growth of dense organo-sedimentary terraces known as ”mattes”^20^. Therefore, secondly, locally enhanced seagrass mortality leads to lower topographic elevation, and vice versa^20^. Third, topographic heterogeneity leads to Bragg reflection^33^, which triggers a bed shear stress pattern^39^, closing the feedback cycle. If the induced bed shear stress pattern and the vegetation density modulation reinforce each other, this feedback mechanism could destabilize homogeneous meadows to spontaneously form patterns. Since Bragg reflection is maximal for topographic perturbations at half the wavelength of the wave forcing, we expect seagrass (and hence bedform) patterning at that wavelength, which in turn maximizes Bragg reflection. Overall, this mechanism may reduce wave energy inside the meadow, providing extra resilience against storms to meadows on the verge of collapse.

In this study, we first analyze observational (Sardinia) and reanalyzed (Mallorca) wave data in more detail, to quantify the relationship between water and seagrass pattern wavelengths. We then derive a mathematical model for the interactions between wave reflection, bed shear stress and seagrass-induced bed topography. We linearize this model, to numerically simulate how a uniform equilibrium can become unstable and develop spatial patterns. Finally, we study wave reflection over the simulated seagrass bedforms. We conclude our study with the implications of our findings for the functioning and resilience of seagrass and other patterned marine ecosystems facing intensifying wave climates, and lessons that can be learned to optimize climate-resilient coastal protection. The model developed here is strongly idealized and serves to support a new hypothesis, which was based on preliminary field observations. Our study aims at raising awareness of this plausible mechanism, and should inspire follow-up research that includes higher-complexity modelling and field campaigns.

## Results

### Field measurements of wave conditions and seagrass patterns

To test our hypothesis of Bragg reflection over seagrass bedforms in wave-dominated environments such as the Mediterranean Sea, we first compare the wavelength $\lambda _n$ of seagrass patterns to the wavelength $\lambda$ of incoming water waves, over a range of water depths *H*. We consider two field sites, i.e. the Bays of Alcúdia and Pollença (Mallorca, Spain, Fig. 1a,b) and the Gulf of Oristano (Sardinia, Italy, Fig. 1c). The Mallorcan bays harbour diverse seagrass communities^46^, of which *Posidonia oceanica* is the dominant species. Earlier studies show that these seagrasses exhibit hexagonal patterns of regularly spaced gaps in the meadow, and quantify the wavelength of this pattern at various locations across the two bays^15^. We use bathymetric data from the Life Posidonia project^46^ to find the corresponding water depths at each seagrass pattern location. For the Gulf of Oristano, the wavelengths of banded seagrass patterns and the corresponding water depths are deduced from previous studies^16^. Details are given in the Methods section.

We use wave gauge data to relate seagrass pattern dimensions to wave forcing. Wave characteristics at shallow offshore locations are obtained from a reanalysis using a phase-averaged wave model^48,49^ for the Mallorcan sites and are deduced from wave observations reported in previous studies for the Sardinian site^47^. For all field locations, we use linear wave theory^31^ to propagate these offshore wave characteristics to the very shallow waters where seagrass patterns are found.

Both for the hexagonal gap patterns^15^ in the *Posidonia* meadows at Mallorca (Fig. 1a,b) and the banded seagrass patterns at Sardinia^16^ (Fig. 1c), the seagrass bedforms have a wavelength that is approximately half the wavelength of the most energetic incoming waves (Fig. 1d). This holds for a wide range of depth values, roughly from 3 to 25m. Because wave conditions at the Bay of Alcúdia are very similar to those at the Bay of Pollença, only the conditions at Pollença are shown in Fig. 1d. The limited availability of high-resolution bathymetric data combined with seabed ecosystem cartography for wave-dominated hydrodynamic environments (as elaborated on in the Discussion section) allowed us to analyze only two field sites (both from the Mediterranean Sea) in this study. Nevertheless, our observations provide a first quantitative support for our hypothesis of pattern formation linked to wave reflection.

### Coupled wave-seagrass model

To test our hypothesis that the observed seagrass patterns could self-organize due to Bragg reflection, we set up a mathematical model. The model equations are given in the Methods section and derived in the “Supplementary Information”. Consistent with most existing models for Bragg reflection over static (fixed in time) seabed modulations^33,40,50^, we consider a two-dimensional domain, defined by sea-to-landward direction *x* and upward direction *z*. Wave forcing is assumed to be perpendicular to the coast, such that along-shore variations (*y*-directed) are neglected (see the illustration in the “Supplementary Information”). Surface gravity waves are described by the surface elevation $\eta (x,t)$ relative to reference level $z=0$ and a velocity potential $\phi (x,z,t)$. The latter is related to *x*- and *z*-directed flow components *u* and *w*, respectively, with $u = \partial _x \phi$ and $w = \partial _z \phi$^31^, where $\partial _x$ is the partial derivative with respect to *x*, etc. In the absence of seagrass, water waves are assumed to travel over a flat horizontal seabed at $z = -h$.

Seagrass covers this bare seabed with density *n*(*x*, *t*). As seagrass traps and binds sediment from the water column^51^, we assume that the actual water depth, *H*(*x*, *t*), equals *h* when no seagrass is present, but decreases linearly with increasing seagrass density. This simplified relationship captures the buildup of bed elevation or, alternatively, the reduction of water depth due to the formation of organo-sedimentary terraces or “mattes” resulting from siltation and interweaving by seagrass rhizomes^20^. Since this process has been observed to lead to mattes as old as 5000 years^52^ and resulting mattes up to 11.7m high^53^, we here assumed a steady, linear increase of bed elevation with coninued seagrass growth. The exact rate of seagrass-induced vertical accretion (parameterized in our model through a topography coefficient, *s*, see the Methods) should be determined in future studies. The spatio-temporal development of seagrass density itself is based on model equations developed in earlier studies^17^. We simplify these equations, to describe seagrass dynamics as a combination of lateral dispersion and local facilitation, competition and net growth or mortality. Since high wave energy limits seagrass survival^45^, we assume in our model that the net seagrass mortality rate $\omega$ increases linearly with wave-induced bed shear stress $\tau _b$. Since seagrass and the associated bed topography evolves much slower than the hydrodynamics, we relate seagrass mortality to the time-averaged bed shear stress. These two mechanisms, i.e. seagrass-induced topographic changes and wave-induced changes in seagrass mortality, give rise to fully coupled wave-topography-seagrass dynamics. Since topographic elevation is a direct function of seagrass density, these dynamics can be described by a set of equations for water surface elevation $\eta (x,t)$, velocity potential $\phi (x,z,t)$ and seagrass density *n*(*x*, *t*). These model equations are explained briefly in the Methods section; the full derivation is given in the "Supplementary Information".

### Uniform model solution and modulation instability

Given that the topographic variations inside seagrass meadows of *Posidonia oceanica* are typically small (order of 1m^54^) compared to typical water depths (order 10–40 m^15,45^), it is expected that the effect of seagrass-induced topography on wave hydrodynamics is relatively small. Following earlier studies^33^, this allows us to separately model the dynamics of a spatially uniform basic state $(\phi _0, \eta _0, n_0)$ and of small spatio-temporal perturbations $(\phi _1, \eta _1, n_1)$ to this basic state. We will first describe how changes in wave forcing affect the basic state, and then show for which forcing conditions the basic state becomes unstable to perturbations, leading to spatial patterning.

**Figure 2:**
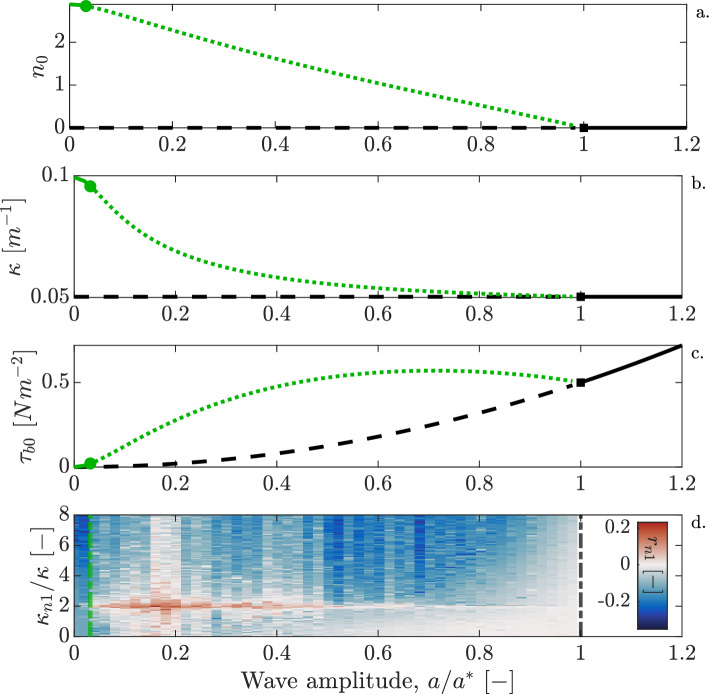
Uniform basic state of the wave-seagrass model and its stability to modulations, as function of wave forcing strength. (**a**–**c**) Numerical solution of the uniform basic state solutions of seagrass density $n_0$, water wavenumber $\kappa$ and bed shear stress $\tau _{b0}$, as a function of forcing water wave amplitude *a*. Wave amplitude is normalized by critical wave amplitude $a^*$, i.e. the value of *a* at the transcritical bifurcation point (black square). The unvegetated state $n_0 = 0$ is indicated in black; the vegetated state $n_0 > 0$ in green. Dashed line indicates instability to homogeneous perturbations, dotted line indicates instability to modulations, and solid lines are stable states. The green circle indicates the modulation instability, whose location ($a = a_{MI}$) is estimated from numerical simulations, i.e. $a_{MI}/a^* \approx 0.03$. (**d**) Dimensionless linear growth rates $r_{n1}$ of the perturbation seagrass state $n_1$, as a function of wavenumber $\kappa _{n1}$ (normalized by forcing water wavenumber $\kappa$) and normalized wave amplitude $a/a^*$. Green and black dash-dotted vertical lines indicate the location of $a = a_{MI}$ and $a = a^*$, respectively. Seagrass perturbation field is imposed on the vegetated basic state ($n_0 > 0$). Growth rates are approximated from the relative growth of $n_1$ after 25 wave periods, as described in the "Supplementary Information". The growth rates show that for $a < a_{MI}$, perturbations decay, whereas $n_1$ becomes modulated with dominant wavenumber ${\kappa _{n1}}^* = 2\kappa$ for $a > a_{MI}$. Values of the model parameters are given in the "Supplementary Information".

The hydrodynamic part of the basic state is given by $\eta _0(x,t)$ and $\phi _0(x,z,t)$. Following some common assumptions (see the "Supplementary Information"), the basic state can be described by a monochromatic linear gravity wave with wave amplitude *a*. Since the hydrodynamic basic state is perfectly periodic, the basic state (time-averaged) bed shear stress $\tau _{b0}$ and, consequently, the basic state seagrass density $n_0$, are constant. Uniform seagrass density is governed by a cubic equation (see derivation in the "Supplementary Information") and therefore has three solutions. We only consider physically realistic solutions, i.e. $n_0 \ge 0$ (Fig. 2). We choose the net mortality rate $\omega <0$ such that, in absence of wave forcing ($a=0$), vegetation grows to form a stable homogeneous seagrass meadow. In the absence of seagrass ($n_0 = 0$), bed shear stress $\tau _{b0}$ increases quadratically with increasing wave amplitude *a* (black line in Fig. 2c), which translates to a quadratic increase of net basic state seagrass mortality $\omega _0$. The presence of a uniform seagrass meadow leads to a spatially uniform buildup of “matte” and thus reduces the homogeneous water depth $H_0$ in a spatially uniform manner. As a result, seagrass-covered seabeds experience a larger shear stress than bare seabeds (green line in Fig. 2c). With stronger wave forcing (larger *a*), increasing bed shear stress enhances seagrass mortality, which reduces vegetation density (Fig. 2a). Hence, the difference in $H_0$ between the vegetated and unvegetated state becomes smaller as *a* increases, up to the transcritical bifurcation point (black square) where $n_0$ becomes zero. The decrease in water depth $H_0$ also results in shoaling of the incoming waves, and thus a decrease in the wavenumber $\kappa$ of the wave forcing (Fig. 2b).

We now discuss the stability of the uniform basic state to perturbations. As derived in the "Supplementary Information", the unvegetated uniform equilibrium $(n_0 = 0)$ is unstable to homogeneous perturbations for $a < a^*$ (dashed black lines in Fig. 2a–c), where $a^*$ indicates a transcritical bifurcation point (black square). For $a > a^*$ the unvegetated state is the only possible uniform equilibrium, which is stable (solid black lines in Fig. 2a–c). For $a < a^*$ a vegetated equilibrium state ($n_0 > 0$) exists. We deduce the stability of the vegetated state by numerically simulating the development of perturbations $(\phi _1, \eta _1, n_1)$ imposed on the vegetated basic state, as explained in the "Supplementary Information". For very weak forcing, $a < a_{MI}$, these perturbations decay with time (Fig. 2d), indicating that the uniform vegetated state is stable (green solid lines in Fig. 2a–c). A modulation instability exists at $a = a_{MI}$. Beyond this threshold, perturbations with a certain wavenumber grow. For the perturbation seagrass density $n_1$, perturbations with wavenumber $\kappa _{n1} = {\kappa _{n1}}^* = 2 \kappa$ show the fastest growth rates (Fig. 2d). This persists until $a = a^*$, although the growth rate of these modulations decreases for *a* increasing beyond $a/a^* \approx 0.5$. In the "Supplementary Information", the trajectories of the spectral power at ${\kappa _{n1}}^*$ as a function of time are also shown, for different values of wave forcing $a/a^*$. These trajectories clearly show that for $a< a_{MI}$, modulations of the vegetation density decay and homogeneous meadows prevail, while for $a > a^*$ vegetation collapses to the bare state. For intermediate values of *a*, however, one would expect periodic patterns with half the wavelength of the forcing.

With increasing coupling strength between wave forcing and seagrass mortality (increasing $\omega _c$; see the Methods), less wave forcing is needed to initiate pattern formation. In other words, $a_{MI}$ decreases with increasing $\omega _c$ (see the "Supplementary Information"). However, the critical wave amplitude $a^*$ also decreases with increasing $\omega _c$, such that the relative position of the modulation instability, $a_{MI} / a^*$ stays almost unchanged.

### Simulated pattern formation and Bragg reflection

**Figure 3:**
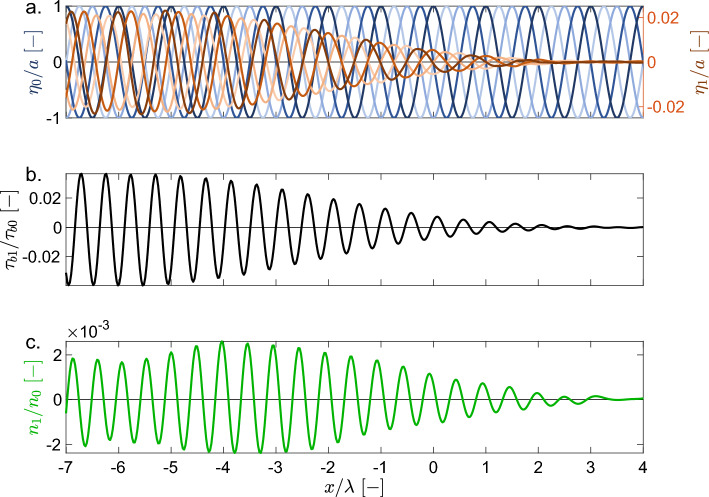
Pattern formation in the wave-seagrass model. (**a**) Incoming wave field (i.e., basic-state water surface elevation $\eta _0$) is shown in blue (darkness increasing over time, i.e. wave travelling to the right) and reflected wave field (i.e., perturbation-state surface elevation $\eta _1$) is shown in orange shades (wave travelling to the left). Water surfaces are shown at four instances within the 25$^{th}$ wave period ($t/T = 24 \frac{1}{4}$, $24 \frac{2}{4}$, $24 \frac{3}{4}$ and 25), with *T* the wave period. Surface elevations $\eta _0$ and $\eta _1$ are normalized by the forcing wave amplitude *a*. (**b**) Perturbation-state bed shear stress $\tau _{b1}$ (normalized by basic-state bed shear stress $\tau _{b0}$), at $t/T = 25$. (**c**) Perturbation-state seagrass density $n_1$ (normalized by basic-state seagrass density $n_0$), at $t/T = 25$. Horizontal direction *x* is normalized by the incoming water wavelength $\lambda = 2\pi /\kappa$. Note that seagrass perturbation $n_1$ develops a periodic structure of periodicity approximately $0.5 \lambda$. The horizontal domain is truncated; for the full domain, including sponge layers, see the "Supplementary Information".

The development of seagrass patterns and resulting topographic modulations goes hand in hand with wave reflection. Interaction between incoming and reflected waves (Fig. 3a) creates a partially standing wave, which yields a partially standing pattern of the perturbation-state bed shear stress $\tau _{b1}$ (Fig. 3b). This modulates the perturbation-state seagrass density $n_1$ (Fig. 3c) and hence the seagrass-induced bed topography. The preferential growth of bedforms with wavenumber ${\kappa _{n1}}^*$ (Fig. 2d) in turn drives Bragg reflection. The seagrass pattern is continuously excited in the simulation domain by the constant wave forcing $(\phi _0, \eta _0)$ and migrates to the left, i.e. facing the incoming waves. The amplitude of the excited wave $(\phi _1, \eta _1)$ increases in amplitude towards the left until it reaches the left sponge layer, where its amplitude is gradually dampened out (see the "Supplementary Information"). As a result, the amplitudes of the bed shear stress and seagrass pattern (and hence the topographic pattern) show a similar profile (Fig. 3b,c).

**Figure 4:**
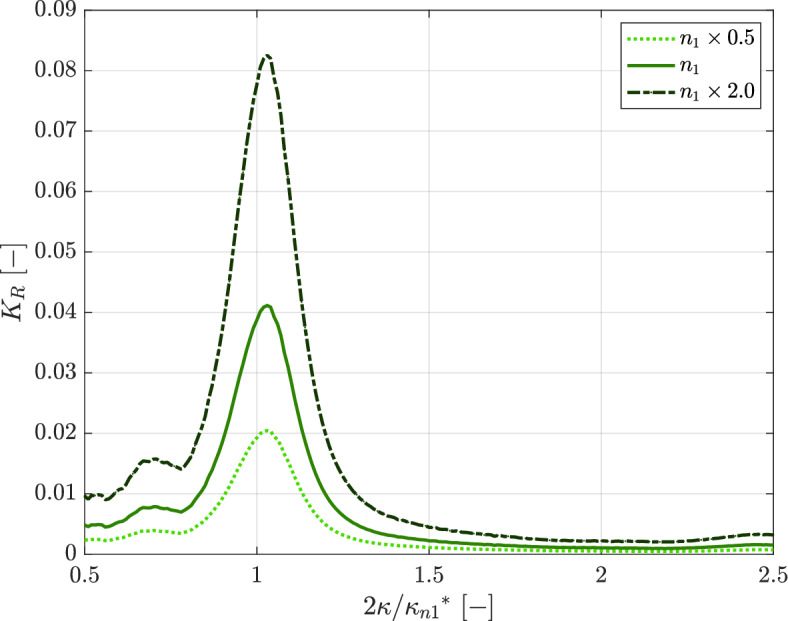
Wave reflection coefficient as a function of the incoming water wavenumber, for different amplitudes of the seagrass pattern. We fix the seagrass pattern $n_1$ and the related topography (with dominant wavenumber ${\kappa _{n1}}^*$) shown in Fig. 3c. A series of simulations is then performed with this fixed topography; each simulation with slightly different wavenumber $\kappa$ of the forcing wave field $(\phi _0, \eta _0)$, as shown along the horizontal axis. Wave reflection coefficients $K_R$ are measured as the maximal absolute value of $\eta _1 / a$ within the interior domain (i.e. excluding sponge layers). $K_R$ is maximal for forcing wavenumber $\kappa = {\kappa _{n1}}^* / 2$, which is equal to the wave conditions under which the pattern of Fig. 3c formed. This implies that self-organized patterns cause a seagrass meadow to reflect away the incoming waves much more efficiently. The calculations are first done with the fixed seagrass pattern from Fig. 3c (middle curve, i.e. solid green line) and repeated with the same seagrass pattern but *halved* in amplitude (lighter green, dashed line) and repeated with this seagrass pattern but *doubled* in amplitude (darker green, dash-dotted line). This shows that the reflection coefficient scales with seagrass pattern amplitude.

Self-organization of the seagrass meadow leads to enhanced wave reflection, as can be seen in Fig. 4. Given the large separation of timescales between seagrass pattern growth and hydrodynamics, we consider that the pattern forms over many years upon averaging the effects of the most energetic wave conditions, while it can be considered fixed on the shorter timescales over which wave forcing varies (e.g., daily to seasonal variability). To simulate the effect of a (fixed) seagrass and bedform pattern on these shorter time-scale hydrodynamics, we fix the simulated bedform pattern (linearly related to $n_1$ via a topography coefficient *s*) of Fig. 3c. We then numerically time-integrate the model equations (wherein only the wave hydrodynamics evolve, since seagrass/topography is fixed), for a series of wave forcing conditions $(\eta _0, \phi _0)$, each with slightly different forcing wavenumber $\kappa$. Figure 4 shows that wave reflection is maximal for the wave conditions that created this seagrass and topographic pattern in the first place (Fig. 3), i.e. waves with $\kappa = {\kappa _{n1}}^* / 2$. These findings agree with the wave reflection coefficients for Bragg-reflecting water waves propagating over fixed sinusoidal sand ripples^33^, hence further supporting our hypothesized seagrass patterning mechanism. Figure 4 implies that the self-organized seagrass pattern shields off the most dominant waves with about a factor 10 more efficiently compared to less dominant wave conditions. Wave reflection increases with the amplitude of the seagrass pattern (in line with findings for fixed bed ripples^33^), which implies that a patterned seagrass meadow becomes more wave-reflecting as its patterns develop over time. Wave reflection efficiency is furthermore expected to grow with increasing extent of the patterned seagrass meadow^33^.

## Discussion

Insight into spatial pattern formation in ecosystems worldwide is crucial to understand how patterning contributes to ecosystem resilience in a changing climate. In this study, we propose that spatially regular patterning in wave-exposed seagrass meadows can be explained at least in part by the interaction of Bragg reflection of ocean waves, bed shear stress-induced seagrass mortality and seagrass-induced topographic build-up. We base our hypothesis on field observations from two field sites in the Mediterranean Sea, which suggest that one-dimensional (banded) and two-dimensional (hexagonal) patterns in *Posidonia oceanica* meadows have a pattern wavelength which is roughly half the wavelength of the most energetic water waves at these locations, over a broad range of water depths. This wavelength relation suggests that Bragg reflection of water waves plays a role. We derived a mathematical model for water wave propagation and reflection coupled to seagrass growth dynamics and resulting topographic changes. Focussing on the onset of seagrass pattern formation, we numerically solve the linearized model equations. This reveals that seagrass meadows remain uniform for weak wave forcing, but that beyond a critical wave forcing strength, the meadow density and the corresponding topography become modulated with wavelength half that of the forcing water waves. Finally, we show that wave reflection varies with changing water wavelengths, but has a steep maximum for water wavelengths twice the meadow pattern wavelength. This finding is consistent with Bragg reflection of water waves over stationary abiotic bed ripples. We thus propose a novel mechanism to explain regularly patterned seagrass patterns in wave-exposed waters such as the Mediterranean, which can possibly be found under similar hydrodynamic conditions in other parts of the world. This may advance our understanding of the influence of patterning on the resilience of ecosystems such as wave-exposed seagrass meadows. Furthermore, our findings suggest that patterning of coastal ecosystems may enhance wave reflection, thus contributing to their coastal protection value.

The observations from two Mediterranean Sea sites of seagrass patterns with a wavelength roughly half that of the wave forcing provide a first indication that Bragg reflection may play a role, and our model findings further support this hypothesis. Previous studies on Bragg reflection of water waves have focussed mainly on Bragg reflection over static bed ripples^33^. Other studies have shown that the partially standing wave pattern at Bragg resonance can create a pattern in bed shear stress and that this may lead to the formation of bedforms^39,40,42,43,55^. Bragg reflection has also been shown to occur over static bedforms with a porous surface and dense arrays of cylinders, both of which can be seen as abstractions of submerged vegetation^56–58^. However, to our knowledge, no studies have previously shown how Bragg reflection can occur in dynamic interactions between wave motion, vegetation growth and bed morphology. Thus, our study proposes a novel mechanism to explain seagrass pattern formation where waves are the dominant physical driver. In many previous studies on self-organization in marine ecosystems, tidal currents are assumed to be the dominant shaping factor^6,7,59^. Waves are usually assumed to be a source of disturbance^19,21^. We show that ocean waves can in fact have a clear shaping influence as well. With increasing wave strength due to global change^29,30^, wave-induced self-organization may become an increasingly important process in marine ecosystems. A similar mechanism might also be important in the patterning of other wave-dominated coastal ecosystems, such as coral and shellfish reefs.

The observational support for our theoretical model was based on two study sites in the Mediterranean Sea (Fig. 1). Follow-up studies should test if similar relationships between the wavelengths of ocean waves and seagrass patterns are found more globally. We have attempted to do such a global analysis, but found that insufficient data is available to test this. In theory, freely available satellite imagery^60^ could be used to detect and quantify the wavelength of seagrass patterns in other wave-dominated seas, which could then be related to global gridded bathymetry data^61^, and the most energetic wave conditions (e.g., wave reanalysis data^32^). However, the water depth penetration of aerial imagery is typically very limited (e.g., about 5 meters for the still relatively clear Mediterranean waters above the seagrasses in Kerkennah, Tunisia), which precludes an assessment of seagrass pattern wavelengths over a significant depth range, which would be needed to further test the preliminary observations in Fig. 1d. Furthermore, aerial images cannot be used to assess whether visually observeable seabed patterns are composed of seagrasses or of other ecosystems with possibly different biogeomorphic effects. Furthermore, the spatial resolution of global gridded bathymetry data^61^ (about 400m horizontal and 1m vertical resolution) is too coarse to accurately quantify the relation between seagrass pattern wavelengths and water depth, especially in shallow coastal regions or areas with steep bed slopes. Therefore, more (sonar-based) surveys such as the ones analyzed in our study^15,16^ are needed.

Apart from the limited availability of such freely available global data, the fact that seagrass patterns can be formed by several alternative processes (of which our newly proposed mechanism may hold in some regions, but not everywhere) makes it difficult to interpret such remotely sensed data without further knowledge of conditions in the field. Analysis of aerial images of seagrass patterns from around Kerkennah (Tunisia) and Shark Bay (Western Australia) did neither provide clear support nor a rejection of our hypothesis, as different types of patterns with diferent dimensions can be observed. E.g., the seagrass patterns around Kerkennah can be seemingly regularly spotted (34.7181$^\circ$N, 11.1462$^\circ$E) or gapped (34.6402$^\circ$N, 10.9848$^\circ$E), but resemble travelling pulses linked to sulfide poisoning^18^ elsewhere (34.8142$^\circ$N, 11.2392$^\circ$E). Also in Shark Bay, different types of patterns with different length scales co-occur. Whereas striped patterns have wavelengths roughly in agreement with our hypothesis in some regions (e.g., a pattern wavelength of around 110m at a depth of about 9m, around 25.8790$^\circ$S, 114.1277$^\circ$E, roughly consistent with the expected 88m given a 99-percentile peak wave period of 19s in deep water, as estimated from wave-reanalysis data^32^), larger-than-expected striped patterns are observed elsewhere (e.g., a pattern wavelength of about 160m at a depth of about 5m, around 25.9608$^\circ$S, 114.0318$^\circ$E, which is larger than the expected 66m). Furthermore, seagrass patterns resemble travelling pulses in other regions (e.g., around 25.8812$^\circ$S, 113.9479$^\circ$E). It is therefore likely that the seagrass patterns around Kerkennah and in Shark Bay (and probably in other regions worldwide) can be formed by multiple (possibly even interacting) different mechanisms. Our proposed mechanism of wave reflection may explain seagrass patterns in some, but not all, wave-dominated environments.

The gapped and striped seagrass patterns found around Mallorca and Sardinia (Fig. 1) have wavelengths that are considerably larger than those expected for patterns formed by sulfide poisoning^18^ and tidal scouring^19^. Why the seagrass patterns in Shark Bay have wavelengths roughly consistent with our Bragg reflection hypothesis in some areas, but are larger than expected in other regions, remains to be investigated. Tidal currents possibly play a stronger role in Shark Bay than they do in the Mediterranean Sea, either interacting with or replacing our proposed Bragg patterning mechanism. Another possibility might be that longer ocean waves, such as infragravity waves generated by tropical cyclones^62,63^ contribute to the formation of these larger-scale banded seagrass patterns, a notion supported by observations of Bragg reflection of infragravity waves^64^. In any case, a more global analysis of seagrass pattern wavelengths to support our hypothesis is beyond the reach of the current study, and an analysis of worldwide aerial imagery alone will likely not suffice, as in-depth seabed mapping is required. In our study, we have therefore limited our analysis to field sites in the Mediterranean Sea, where wave forcing dominated over tidal forcing, and for which detailed seabed cartography and bathymetric surveys were available.

Our study assumes that biotic interactions, i.e. seagrass-induced bed structuring, form a crucial part of the feedback cycle that leads to seagrass patterning. The field observations from Sardinia and Mallorca considered in our study (Fig. 1) are from environments where seagrass is densely covering the seabed and thus strongly affects bed morphology^15,16^ through enhanced sediment trapping, interweaving with rhizomes and the formation of dense organo-sedimentary “mattes”^20,52,53^. We hypothesize that the interactions between Bragg reflection and seagrass dynamics can explain the regular patterns observed in these dense meadows, and we support this with an idealized model. Our study does not preclude the possibility, however, that the observed bedforms are initially induced by abiotic processes. As mentioned above, Bragg reflection can also lead to the formation of bed ripples in the absence of vegetation^39,40,42,43,55^. In theory, banded seagrass patterns could therefore be the result of physically formed bedforms, where seagrass later (passively) colonizes bedform crests rather than troughs. In fact, the relative importance of abiotic and biotic processes in the formation of such bedform patterns will likely differ from site to site, ranging from bedforms generated purely abiotically^39,40,42,43,55^, to abiotically generated bedforms that are passively colonized by seagrass^65^, to bedform patterns in meadows characterized by thick matte formation^15,16,20,52,53^. The development of an objective “indicator” for this relative biotic-vs-abiotic importance in bedform generation, as was previously done for the relative importance of biofilms for bedform creation on intertidal flats^3,66^ should be the scope of future research. Field manipulation experiments^51^ and more realistic numerical modelling studies that couple seagrass-dynamics^15^ with realistic sediment and hydrodynamics^40^, will be essential to develop such indicators.

However, based on the geometric properties of the observed bedforms (Fig. 1), we can already assess that it is likely that seagrass plays a considerable role in their formation. Firstly, the banded seagrass patterns observed in the field have relatively larger steepness (ripple height over wavelength) of 0.08–0.12^16^, compared to observed sand ripples induced by Bragg resonance with steepness of 0.01–0.07^55^. Steep abiotic ripples (ranging from 0.07^39^ to 0.18^43^) have been generated due to Bragg reflection in the lab, but it remains the question whether lab-generated bedform characteristics are one-to-one comparable with real-world (field) conditions. Hence, although bed ripples of similar dimensions could be formed without vegetation as well, the relatively high bedform steepness in seagrass meadows at least suggests that vegetation plays an important role in bedform construction. Similarly, bedform steepness is typically considered a leading indicator of the relative importance of biofilms for bedform creation on intertidal flats^3,66^. Furthermore, the observation of regular hexagonal seagrass patterns with the same relation between pattern and water wavelengths^15^ (Fig. 1) suggests that abiotic processes (sand bar formation) alone cannot explain the patterns in this particular site. Hexagonal patterns are a typical two-dimensional generalization of one-dimensional regular banded patterns due to quadratic nonlinear interactions among plants^44^. Bragg reflection has also been demonstrated before for the propagation of water waves over two-dimensional regular patterns^34^. To our knowledge, hexagonal symmetry is not typical for purely abiotically generated sandbars. In conclusion, the processes of bedform patterns in environments with seagrass can range from purely abiotic through biotically-mediated to biotically dominated. The one-dimensional banded seagrass patterns^16^ considered in our study could in principle be formed without active involvement of biota, but the relatively high ripple steepness, in combination with observations of two-dimensional (hexagonal) seagrass patterns^15^ provides clear support for our hypothesis that biogeomorphic interactions play an important role in the patterning of these wave-exposed seagrass ecosystems.

The seagrass patterns in our model seem to arise due to a noise-sustained convective instability. Small perturbations to the uniform seagrass meadow grow (initially) linearly over time, and migrate against the direction of wave forcing, i.e. convective instability. Continuous wave forcing is required to keep triggering this convective instability, and hence this process can be regarded as noise-sustained. This class of instabilities has been reported earlier for magnetic fields^67^, fluid dynamics^68,69^ and optics^70^. To our knowledge, noise-sustained convective instabilities have not received much attention yet in the context of ecosystem dynamics, possibly because their extremely slow motion would require decades to be observed. Seagrass patterns have been reported to migrate in the field as well, both with seaward^71^ and landward^65^ migration directions. Whether the simulated seaward direction of bedform migration and the rate of migration are realistic, requires further study.

Our research focuses on the initiation of seagrass pattern formation. Although we derive the fully nonlinear model equations (see the Methods) for wave-seagrass-topography interactions, we linearize the hydrodynamic equations to facilitate their numerical solution (see the "Supplementary Information"). Since we can assume that, in the real world, the amplitude of seagrass-induced bedforms always remains much smaller than the water depth, we do not linearize the seagrass equation. Although we expected that the nonlinear mortality term in the seagrass equation would cause the seagrass pattern to reach an equilibrium state, the simulated seagrass pattern does not reach such equilibrium. Instead, the seagrass pattern migrates in the direction opposite to the incoming waves and reaches the sponge layer before its amplitude saturates. This migration may be explained either because the phase of the bed shear stress pattern is slightly shifted relative to the bedform pattern^33^, and/or because the partial reflection of the incoming wave results in a partial standing wave, which itself migrates over time. Furthermore, the amplitude of the simulated pattern grows, saturates and then oscillates with a period much longer than the wave period ("Supplementary Information"). This “beating” of the wave envelope could be a result of reflection of the perturbation wave field $(\phi _1, \eta _1)$ against the lateral domain boundaries. This is not expected, however, since the employed sponge layer is highly effective in damping away the perturbation wave field. Most probably, interactions between nonlinear seagrass dynamics and the sponge layers give rise to these long-term oscillations. Since we think these are spurious dynamics caused by the boundary conditions of our simulations, we choose to restrict our attention to the onset of pattern formation, i.e. the stage of pattern formation where the linearization assumptions of small bedform amplitude ("Supplementary Information") are still valid. For the purpose of our study, which is to demonstrate that Bragg reflection could explain the observed seagrass patterns, the restriction of our simulations to this initial, linear regime is sufficient.

Although the linearization serves the purpose of this study, some model assumptions are crude and could benefit from refinement in future studies. First, by performing a series expansion and subsequently linearizing the wave equations (following earlier studies^33^), it is implicitly assumed that partial reflection of the incoming wave over the rippled seabed does not reduce the energy of the wave further down. For the relatively small bedform amplitudes considered in the linear regime of this study, this assumption does not significantly affect the outcome. However, to simulate the formation and equilibration of seagrass patterns with relatively larger amplitude, this assumption may become less realistic. Furthermore, when seagrass-induced bedforms have a significant effect on wave reflection, this will also result in a reduction of the transmitted wave energy. Therefore, a logical next step would be to solve the full, nonlinear set of equations (see the Methods). Lateral boundary conditions (sponge layers) for the nonlinear wave equations have been derived earlier for fixed bed ripples^72^, but the time-dependence (growth and migration) of the bottom boundary in our case complicates this situation and may require sponge layer functions that adapt to this change in bed topography. Full nonlinear simulations of our model equations in larger domains, to avoid spurious boundary effects and properly resolve large amplitude patterns, should be addressed in future studies.

Further model extensions can be made to obtain more realistic model predictions, however we do not expect that this will qualitatively change our principal findings. Firstly, calibration of the model parameters based on observations will lead to quantitatively more realistic predictions. In particular, the topography coefficient *s* (which linearly translates seagrass density to seabed topography) and the sensitivity of seagrass mortality to bed shear stress ($\omega _c$) determine the coupling strength between wave motion and seagrass-induced topographic changes. Secondly, relaxing the assumption of an ideal fluid and including the effects of turbulence and bottom friction on the flow will increase the accuracy of the hydrodynamic simulations. However, Bragg reflection is also found with wave models that resolve all these hydrodynamic details^16,73^ and for waves propagating over dense arrays of cylinders^58^ or porous beds^56,57^ (both representative of the effects of submerged vegetation). This suggests that the main findings of our study will remain, even when using more detailed and calibrated models. Here we have chosen a highly idealized approach that allows us to identify a novel ecosystem-patterning mechanism.

Our study suggests that self-organized patterning of seagrass meadows strongly increases the wave reflecting efficiency of these marine ecosystems. Field measurements are needed to verify this theoretical finding. In particular, direct measurements of wave reflection over patterned seagrass meadows are required, e.g., following the approach taken by^38^. Simultaneous measurements over seagrass meadows that are spatially more uniform^28^ should be performed as a control. Such a series of control measurements should separate the effects of meadow gap/band patterns (i.e., the Bragg reflection showed in our simulations) from the confounding wave reflection signal induced by the raised elevation and roughness of the meadow in its entirety^74^.

The potential implications are two-fold. First, it suggests that this self-organization process helps protect the meadow further down-wave against wave stress, which might increase seagrass resilience against deteriorating environmental conditions (e.g., increasing wave power^29,30^). This implication is in line with recent findings that spatial patterns bolster ecosystem resilience^13^. Since the patterns in our simulations do not reach an equilibrium state, we were not able to compute the equilibrium seagrass densities of the patterned state and compare these to the uniform seagrass densities in Fig. 2a. If an equilibrium pattern state is achieved in future model extensions, such a comparison can confirm whether seagrass patterning indeed increases ecosystem resilience to wave stress. A second implication is that wave reflection in patterned marine ecosystems may enhance the coastal protection of densely populated coastlines. Whereas seagrasses and other marine vegetatated ecosystems are well-known to dampen wave energy^26,28,75^ and meadow gaps are known to enhance turbulent kinetic energy dissipation^76^, the effect of meadow patterning on (Bragg) wave reflection has (to our knowledge) not been well studied yet. Furthermore, wave reflection may also be an important effect in other patterned marine ecosystems such as coral and shellfish reefs^5,6^, as well as tidal flats and marshes^3,4^. Whereas undulating artificial bars (breakwaters) have already been used to enhance coastal protection via Bragg reflection^36,50,77,78^, our study suggests that ecosystems may naturally have these protective characteristics. This calls for further investigation of the use of natural and restored coastal ecosystems to strengthen coastal protection against the flood risks that increase with climate change^29,30^.

## Methods

### Analysis of field data

For the Bays of Alcúdia and Pollença (Mallorca, Spain), wavelengths of the hexagonal seagrass gap pattern are obtained from literature^15^, while corresponding water depths are deduced from Life Posidonia bathymetric data^46^. To obtain realistic wave data for these bays, we use reanalysis data from a phase-averaged wave model^48,49^. The model solves the energy-balance wave equation, thus advecting the energy spectra from deep to shallower waters. A. Orfila provided the wave data for the Bays of Alcúdia and Pollença, which were calculated by Luque et al.^49^ from the openly accessible deeper-water wave data from Mentaschi et al.^48^. We consider a time series of wave data from 1980 to 2016, at a measurement point in Pollença Bay (point 20, 39.9002$^\circ$N, 3.1480$^\circ$E, depth $H = 29.7$ m) and Alcúdia Bay (point 53, 39.7956$^\circ$N, 3.2320$^\circ$E, depth $H = 29.8$ m). From these time series, wave spectra are calculated per 3-hour interval. For each of these spectra, significant wave height (mean height of the 33% highest waves) and peak wave period (the wave period of the highest-energy waves) are calculated. The corresponding water wavelengths $\lambda$ for each 3-hour wave spectrum can be calculated using linear wave theory^31^. The dispersion relation is given by

1 $$\begin{aligned} \sigma ^2 = g \kappa \tanh {(\kappa H)}, \end{aligned}$$

with angular frequency $\sigma = 2 \pi / T$, gravitational acceleration *g*, wavenumber $\kappa = 2 \pi / \lambda$ and water depth *H*. Since we are interested in the most energetic waves only, we choose wave periods *T* equal to the peak wave periods obtained from the wave spectra. Using this relation, the time series of *T* at the two measurement locations with known depths *H* can be solved numerically to obtain a time series of wavelengths $\lambda$. The wave-induced bed shear stress $\tau _b$ associated with each wave can be computed from

2 $$\begin{aligned} \tau _b = \frac{1}{2} \rho f_w {U_b}^2, \end{aligned}$$

with water density $\rho$, wave friction factor $f_w$ (assumed to be constant) and near-bed orbital velocity amplitude ${U_b}$ given by

3 $$\begin{aligned} U_b = 2 \pi A_b / T, \end{aligned}$$

with maximum near-bed orbital amplitude $A_b$ and significant wave height $H_s$, i.e.

4 $$\begin{aligned} A_b = \frac{H_s}{2 \sinh {(\kappa H)}}. \end{aligned}$$

Since we are interested only in those waves that most strongly impact the sea bed, from the reanalyzed time series of peak wave periods we only select those events that exert the highest bed shear stress. That is, after converting peak wave periods to $\tau _b$, we select the 0.99 quantile of the distribution of $\tau _b$ (i.e., the $1\%$ of the distribution with the highest bed shear stress). We thus obtain a distribution of the wavelengths $\lambda$ associated with these most energetic waves in Pollença and Alcúdia Bays. Employing the common assumption^31^ of stationarity (i.e., wave properties such as $\sigma$ do not change while a wave travels from deep to shallow waters), we can rewrite dispersion relation (1) as

5 $$\begin{aligned} \frac{\kappa _\infty }{\kappa } = \tanh (\kappa H), \end{aligned}$$

where subscript $\infty$ denotes wave conditions in deep water^31^, i.e. where $\kappa _\infty H_\infty>> 1$. We numerically solve this shoaling relationship for $\lambda$ as a function of *H*, to calculate how the wavelengths of the most energetic $1\%$ of waves decrease as these waves propagate into shallower waters (Fig. 1d).

As a second location, we consider the banded seagrass patterns at the Gulf of Oristano (Sardinia, Italy). Seagrass pattern wavelengths and corresponding water depths from the study by Coppa et al.^16^ were kindly provided by the authors of that study. The data consists of seagrass pattern wavelengths calculated in the 151 grid cells (of 200x200m each) where rhythmic features were present, and the corresponding mean water depth in each grid cell. We use wave conditions measured at the wave gauge at Alghero ($40.5333^\circ$N, $8.1000^\circ$E), which are $H_s = 8$ m, $T = 10$s and wave direction of $308^\circ$ (northwest)^47^. During these so-called Mistral events, the wave propagation direction is approximately perpendicular to the banded seagrass patterns in the north of the Gulf of Oristano^16,47^. We use dispersion relation (1) to calculate the typical wavelength of such Mistral-induced waves at the location of the wave gauge ($H = 94$ m, data from EMODnet^79^) and propagate these waves to the shallower waters of the Gulf of Oristano using Eq. (5), see Fig. 1d.

### Wave-seagrass model

#### Model setup

In this study, we derive a fully coupled wave-seagrass model. In the current Methods section, we briefly describe the main model characteristics. A full derivation of these equations is given in the "Supplementary Information". In our model, the vegetation density of seagrass covering the bare seabed is given by *n*(*x*, *t*). As seagrass efficiently traps and binds sediment from the water column^51^, it reduces the constant water depth *h*. We assume that the actual water depth, *H*(*x*, *t*), is linearly related to seagrass density, i.e.

6 $$\begin{aligned} H(x,t) = h - s \ n(x,t), \end{aligned}$$

with constant topography coefficient *s*. This parameter accounts also for the living part of the plant, as rhizomes interweave the trapped sediment to form terraces or “mattes” of up to several meters high^20,52,53^, effectively reducing water depth for wave propagation^20^. The topography coefficient *s* therefore defines the rate or efficiency with which seagrass growth leads to vertical buildup of bed elevation. The important effect of seagrass canopy on friction and flow attenuation^28,75,80,81^ is not considered in our theoretical study, in order to isolate the effect of seagrass on wave reflection. Wave damping should be included in follow-up research. The spatio-temporal development of seagrass density is given by a simplified version of an earlier model^17^, i.e.

7 $$\begin{aligned} \partial _t n(x,t) = -\omega n + \alpha n^2 - \beta n^3 + \delta \partial _{xx} n \qquad \qquad \text {at } z = -H, \end{aligned}$$

with net mortality rate $\omega$, facilitative and competitive interaction coefficients $\alpha$ and $\beta$, respectively, and dispersion coefficient $\delta$. Field measurements show that seagrass survival is limited by high wave energy^45^. We incorporate this effect as a linear increase of seagrass mortality with wave-induced time-averaged bed shear stress $\tau _b$, i.e.

8 $$\begin{aligned} \omega = \omega _b + \omega _c \, \tau _b, \end{aligned}$$

where $\omega _b$ is the constant background value of the net mortality rate and $\omega _c$ represents the coupling strength between bed shear stress, given by (2), and seagrass mortality.

Since the seagrass dynamics is much slower than the hydrodynamics, $\tau _b$ in (8) depends on *time-averaged* flow conditions described by $U_b$, rather than the instantanous flow conditions. That is,

9 $$\begin{aligned} U_b(x,t) =&\sqrt{\frac{\sigma }{\pi }\int _{t-\frac{2\pi }{\sigma }}^{t} \left( \partial _x \phi \right) _{(x,-H,t')}^2 \ dt'}, \end{aligned}$$

where, as we force our model with a perfectly periodic forcing, averaging over one wave period is enough. The subscript $_{(x,-H,t')}$ indicates that the integrand is evaluated at depth $z=-H$ and integrated over the dummy time variable $t'$. Hence, by coupling seagrass mortality in Eq. (8) directly to the time-averaged flow conditions in Eq. (9), we are able to bridge the large gap between the short hydrodynamic time-scale and the long biogeomorphic timescale. This approach is effectively similar to scaling up the morphological change after each hydrodynamic timestep with a so-called morphological acceleration factor, a common method in (bio)geomorphic modelling^82^.

Wave propagation is described by the continuity equation, written as the Laplace equation, i.e.

10 $$\begin{aligned} \partial _{xx} \phi + \partial _{zz} \phi&= 0{} & {} \qquad \qquad \text {in } {\mathscr {D}}, \end{aligned}$$

where ${\mathscr {D}}$ denotes the entire fluid domain, i.e. $|x|<\infty , -H \le z \le \eta$. The dynamic boundary condition at the water surface is given by the Bernoulli equation, i.e.

11 $$\begin{aligned} \partial _t \phi&+ \frac{1}{2} \left( (\partial _x \phi )^2 + (\partial _z \phi )^2 \right) + g \eta = 0{} & {} \qquad \qquad \text {at } z = \eta . \end{aligned}$$

The kinematic boundary condition at the water surface is given by

12 $$\begin{aligned} \partial _z \phi&= \partial _t \eta + \partial _x \phi \ \partial _x \eta{} & {} \qquad \qquad \text {at } z = \eta . \end{aligned}$$

Finally, the kinematic boundary condition at the bottom (which is either a flat and bare seabed or the top of a seagrass-covered and hence topographically modulated sea floor) is given by

13 $$\begin{aligned} \partial _z \phi&= s ( \partial _t n + \partial _x \phi \ \partial _x n ){} & {} \qquad \qquad \text {at } z = -H. \end{aligned}$$

Together, Eqs. (7) and (10)–(13) describe the coupled spatio-temporal dynamics of seagrass-induced bed topography and waves that propagate and reflect over this bed topography, which in turn affects seagrass growth. The full derivations of these equations are given in the "Supplementary Information". Note that we did not take into account the effects of bottom friction on the flow or the effect of seagrass canopy on wave damping, which has been shown to be highly important^28,75,80,81^. This simplification allowed to study in isolation the effect of meadow self-organization under Bragg reflection. Future studies should, however, include the viscous effects of frictional wave damping^83^, for a more complete understanding.

#### Uniform basic state and spatial modulations

Given the relatively small amplitude of seagrass bedforms compared to water depth^15,45,54^, we can expand the velocity potential, $\phi$ into a basic state $\phi _0$ which is unaffected by bed modulations, and small perturbations $\phi _1$, $\phi _2$ etc. This is consistent with earlier studies on wave reflection over small-amplitude fixed seabed modulations^33^. Formally, $\phi = \phi _0 + \phi _1 + \phi _2 + ...$, with $\phi _{m+1} / \phi _m<< 1$. A similar expansion can be performed for the other state variables, $\eta$ and *n*. The full set of coupled wave and seagrass equations can hence be written as a set of equations for the basic state, $(\phi _0, \eta _0, n_0)$ and a separate set of equations describing the leading-order perturbation state, $(\phi _1, \eta _1, n_1)$. As the perturbation equations are fully linear and growth would hence never saturate to reach a quasi-steady pattern, nonlinear facilitation and competition terms are included in the equation for $n_1$ to allow for saturation of seagrass growth and hence equilibration of the wave, seagrass and topographic pattern. The detailed derivation of the basic and perturbation state equations is given in the "Supplementary Information".

Adopting common assumptions from linear wave theory^31^, the hydrodynamic basic state can be expressed analytically as

14 $$\begin{aligned} \eta _0&= a \cos (\kappa x - \sigma t), \end{aligned}$$

15 $$\begin{aligned} \phi _0&= \frac{\sigma a}{\kappa } \frac{\cosh \left[ \kappa (z+H_0) \right] }{\sinh \left[ \kappa H_0 \right] } \sin (\kappa x - \sigma t), \end{aligned}$$

16 $$\begin{aligned} \sigma ^2&= g \kappa \tanh \left[ \kappa H_0 \right] , \end{aligned}$$

i.e. a linear gravity wave with amplitude *a*, travelling over a seabed covered by a homogeneous meadow whose top is at constant depth $H_0 = h - s \ n_0$. We note that, for spatially uniform seagrass meadow/topography, bed shear stress is also uniform due to the averaging in (9). Basic seagrass state $n_0$ can therefore be solved semi-analytically from the steady and spatially uniform version of Eq. (7).

Linear stability of the unvegetated uniform equilibrium ($n_0 = 0$) is straightforward, as it is not coupled to the hydrodynamics, and it can be analyzed analytically (see "Supplementary Information"). For the vegetated uniform equilibrium ($n_0 > 0$), linear stability analysis is not trivial, since this equilibrium becomes unstable to modulations that couple to the time-dependent basic hydrodynamic state. Therefore, we choose to solve the evolution of the linear perturbation equations numerically, as will be discussed hereafter and in the "Supplementary Information". The value of the forcing parameter (wave amplitude *a*) where the vegetated uniform equilibrium becomes unstable to modulations (i.e., where the perturbation field starts to grow linearly) identifies the modulation instability. This critical value, $a_{MI}$, is determined from the numerical simulations.

#### Numerical integration

Since the basic state does not depend on higher-order perturbation states, $(\phi _0, \eta _0, n_0)$ can be solved semi-analytically. The forcing wave field, $(\phi _0, \eta _0)$ is known (14–16) and can hence be imposed for each time *t* and position (*x*, *z*), after which $n_0$ is calculated from the steady and space-independent version of (7). The equations that govern perturbation state variables $(\phi _1, \eta _1, n_1)$, however, need to be solved numerically by spatial discretization and time integration. Owing to the series expansion explained in the previous paragraph, free surface boundary conditions can be prescribed at fixed reference level $z=0$ instead of $z = \eta (x,t)$ and bottom boundary conditions can be applied at fixed depth $z = -h + s \ n_0$ instead of at $z = -H(x,t)$. Thanks to this simplification, the fluid domain ${\mathscr {D}}$ (see illustration in the "Supplementary Information") can be replaced by a rectangular fluid domain ${\mathscr {D}}_0$, which vertically ranges from $z = -H_0$ to $z=0$. A channel of infinite horizontal extent is numerically mimicked by adopting so-called “sponge layers”^72,84,85^ in front of the lateral inflow and outflow boundaries. Within these sponge layers, an *x*-dependent damping coefficient is applied to the perturbation velocity potential $\phi _1$ and surface elevation $\eta _1$. The damping coefficients are zero at the interior border of the sponge layers ($x = -(L - \Delta L)$ for the left sponge layer and $x = L - \Delta L$ for the right sponge layer, with sponge layer width $\Delta L$) and smoothly increase towards the outer edge of the simulation domain ($x = \pm L$). This approach ensures that the perturbation wave field vanishes towards the lateral edges of the simulation domain and does not “feel” the presence of the sponge layer in the interior fluid domain. Numerical solution methods are described in more detail in the "Supplementary Information".

#### Analysis of model results

To determine the dominant wavelength of the simulated seagrass patterns, Fourier spatial power spectra are computed. Fast Fourier Transforms are applied to the seagrass density field $n_1(x,t)$ outside the sponge layers, i.e. for $|x| < L - \Delta L$. Linear stability of the basic state is then computed by measuring the linear growth rate of the fastest growing spectral component of $n_1$. Finally, to study how seagrass pattern formation affects wave reflection, the amplitude of the reflected wave $\eta _1$ is quantified for different ratios of forcing water wavenumber $\kappa$ to dominant pattern wavenumber ${\kappa _{n1}}^*$. First, one simulation is run with wave forcing wavenumber $\kappa$, until a quasi-steady seagrass pattern with dominant wavenumber $\kappa _{n1} = {\kappa _{n1}}^* = 2 \kappa$ has emerged (Fig. 3). The resulting seagrass-induced topography, $-h + s \ n_1(x,t)$, is fixed in time, and a series of new simulations is run, where only the water wave dynamics are computed while seagrass dynamics and hence morphodynamics are fixed. For each subsequent simulation, the forcing wavenumber $\kappa$ is slightly changed while the seagrass pattern and hence ${\kappa _{n1}}^*$ remains the same. Thus, wave reflection is quantified as a function of the ratio $2\kappa /{\kappa _{n1}}^*$. Hydrodynamic simulations are run until the perturbation wave field reaches dynamic equilibrium, after which reflection coefficients $K_R$ are measured. Methodological details are given in the "Supplementary Information".

### Supplementary Information

Supplementary Information.

## Data Availability

Data and scripts can be found under https://doi.org/10.20350/digitalCSIC/15667.
