# Supplementary material for: Optimal wave reflection as a mechanism for seagrass self-organization
Source: Sci Rep. 2023 Nov 20;13:20278. doi: 10.1038/s41598-023-46788-4 (PMC10662035; doi:10.1038/s41598-023-46788-4)
Supplement: Supplementary file 1 — Supplementary Information. [file 41598_2023_46788_MOESM1_ESM.pdf]

Optimal wave reflection as a mechanism for seagrass self-organization: *Supplementary Information*

Roeland C. van de Vijsel[1,2,*], Emilio Hernández-García[1], Alejandro Orfila[3], and Damià Gomila[1]

[1]IFISC (CSIC-UIB). Institute for Cross-Disciplinary Physics and Complex Systems, E-07122 Palma, Mallorca, Spain
[2]Now at: Hydrology and Environmental Hydraulics Group, Wageningen University, The Netherlands
[3]IMEDEA (CSIC-UIB). Mediterranean Institute for Advanced Studies, E-07190 Esporles, Mallorca, Spain
[*]roeland.vandevijsel@wur.nl

ABSTRACT

Supplementary Information for "*Optimal wave reflection as a mechanism for seagrass self-organization*".

Derivation of model equations

Here, the model equations are derived. First, the full set of coupled partial differential equations for wave hydrodynamics and seagrass dynamics will be treated. Then, a series expansion is performed to obtain separate equations for the basic state and for small perturbations relative to this basic state. Finally, semi-analytical solutions for the basic state are described and a linear stability analysis is performed.

Full nonlinear equations

To describe surface water wave motion, we adopt several commonly applied idealizing assumptions[1]. Waves are assumed to propagate through an ideal fluid, i.e. water that is incompressible, has constant density and no viscosity. Gravitation is the driving force, while water surface tension, wind forcing and bottom friction are ignored. Water motion is furthermore assumed to be irrotational. We consider wave motion as a function of time t, in three-dimensional space with horizontal directions x, y and vertical coordinate z (Supplementary Figure S1). The water surface is at elevation $z = \eta(x,y,t)$, which is the deviation from reference elevation $z = 0$. The bottom boundary is located at $z = -H(x,y,t)$. This boundary equals the flat and bare seabed in the absence of seagrass, or the top of the seagrass-covered bed in the presence of seagrass.

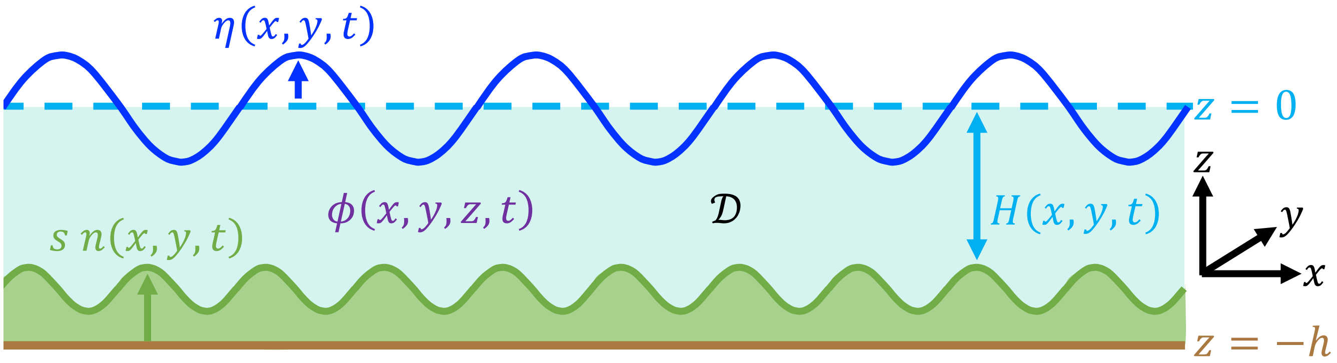

Supplementary Figure S1. Sketch of the domain of interest and state variables ϕ, η and n.

Derivation of the following equations (1) - (5) is well-known[1]. For irrotational fluids, a velocity potential function $\phi(x,y,z,t)$ can be introduced, i.e.

$$\vec{u} = \vec{\nabla}\phi, \tag{1}$$

where $\vec{u} = (u,v,w)$ are the (x,y,z)-components of the water particle velocity and $\vec{\nabla} = (\partial_x, \partial_y, \partial_z)$. In this study, $\partial_x, \partial_y, \partial_z$ and ∂_t denote derivatives with respect to x, y, z and t. The mass balance or continuity equation can now be written as the Laplace

equation,

$$\vec{\nabla}^2\phi = 0, \tag{2}$$

and the dynamic boundary condition at the water surface is given by the Bernoulli equation,

$$\partial_t\phi + \frac{1}{2}\left(\vec{\nabla}\phi\right)^2 + g\eta = 0, \tag{3}$$

with g the gravitational acceleration. Here, atmospheric pressure was assumed to be constant and set equal to zero at the water surface, for convenience. The kinematic boundary condition at the water surface is given by

$$\partial_z\phi = \partial_t\eta + \vec{\nabla}_h\phi\cdot\vec{\nabla}_h\eta, \tag{4}$$

with horizontal gradient operator $\vec{\nabla}_h = (\partial_x, \partial_y)$. Finally, the kinematic boundary condition at the bottom is given by

$$\partial_z\phi = -\partial_t H - \vec{\nabla}_h\phi\cdot\vec{\nabla}_h H. \tag{5}$$

We assume here that the location of the bottom boundary, $z = -H$, is linearly related to dimensionless seagrass density $n(x,y,t)$, i.e.

$$H = h - sn, \tag{6}$$

where h is the constant water depth in the absence of seagrass (i.e., the location of the flat seabed) and s can be interpreted as a topography coefficient, translating seagrass density to topographic elevation. This parameter defines the rate or efficiency at which the seagrass meadow raises the seabed through the formation of rhizome-interwoven sediment deposits or "matte"[2]. Its value is the bed elevation gained due to the meadow when the (dimensionless) seagrass density is of order 1. Its exact value (see Supplementary Table S1) should be calibrated in follow-up studies, but is here chosen consistently with the observation that "mattes" can continue to develop up to more than 10m in height[3,4]. We furthermore assume that dimensionless seagrass density is governed by

$$\partial_t n = -\omega n + \alpha n^2 - \beta n^3 + \delta\vec{\nabla}_h^2 n, \tag{7}$$

with net seagrass mortality rate ω (death rate minus growth rate), facilitative interaction coefficient α, competitive interaction coefficient β and lateral plant dispersion coefficient δ (all dimensionless). Equation (7) was introduced earlier[5]; here we have ignored non-local interactions between plants and plant dispersion due to clonal growth.

We assume that wave-induced bed shear stress $\tau_b(x,y,t)$ exerted on the bottom boundary increases seagrass mortality, i.e.

$$\omega = \omega_b + \omega_c\,\tau_b, \tag{8}$$

where ω_b is the constant background value of the net mortality rate and ω_c represents the coupling strength between bed shear stress and seagrass mortality. Following earlier studies[6–8], we express wave-induced bed shear stress as

$$\tau_b = \frac{1}{2}\rho f_w|\vec{U}_b|^2, \tag{9}$$

with (constant) water mass density ρ, horizontal near-bed orbital velocity amplitude $|\vec{U}_b| = \sqrt{U_b^2 + V_b^2}$ and wave friction factor f_w. For simplicity, we assume f_w is constant. Assuming that the horizontal orbital velocity is a sine wave with period $T = 2\pi/\sigma$ (with angular wave frequency σ), the horizontal velocity amplitude equals $\sqrt{2}$ times the root mean square of the horizontal orbital velocity. In that case,

$$|\vec{U}_b|(x,y,t) = \sqrt{\frac{\sigma}{\pi}\int_{t-\frac{2\pi}{\sigma}}^{t}\left(\vec{\nabla}_h\phi\right)^2_{(x,y,-H,t')}\,dt'}. \tag{10}$$

Then, the full equations for coupled water wave and seagrass dynamics are

$$\vec{\nabla}^2\phi = 0 \qquad \text{in } \mathscr{D}, \tag{11}$$

$$\partial_t\phi + \frac{1}{2}(\vec{\nabla}\phi)^2 + g\eta = 0 \qquad \text{at } z = \eta, \tag{12}$$

$$\partial_z\phi = \partial_t\eta + \vec{\nabla}_h\phi\cdot\vec{\nabla}_h\eta \qquad \text{at } z = \eta, \tag{13}$$

$$\partial_z\phi = s(\partial_t n + \vec{\nabla}_h\phi\cdot\vec{\nabla}_h n) \qquad \text{at } z = -H, \tag{14}$$

$$\partial_t n = -\omega n + \alpha n^2 - \beta n^3 + \delta\vec{\nabla}_h^2 n \qquad \text{at } z = -H, \tag{15}$$

where $\mathscr{D}$ denotes the entire fluid domain, i.e. $|x| < \infty, |y| < \infty, -H \leq z \leq \eta$ (Supplementary Figure S1), with H as in (6) and ω as in (8) - (10). Equations (11) - (15) describe the spatio-temporal dynamics of the state vector $\vec{\chi} = \{\phi, \eta, n\}$.

Series expansion

Inspired by earlier studies[9], we assume that system state $\vec{\chi}$ can be written as a series expansion,

$$\begin{aligned} \phi &= \phi_0 + \phi_1 + \phi_2 + ..., \\ \eta &= \eta_0 + \eta_1 + \eta_2 + ..., \\ n &= n_0 + n_1 + n_2 + ..., \end{aligned}$$

where $|\phi_m|, |\eta_m|, |n_m| = \mathscr{O}(\varepsilon^m)$ and ε is a small and constant scaling parameter, $|\varepsilon| << 1$.

Substitution of the above series expansion in full equations (11) - (15) yields $\mathscr{O}(1)$ equations for the basic state $\vec{\chi}_0$, i.e.

$$\vec{\nabla}^2 \phi_0 = 0 \qquad \text{in } \mathscr{D}, \tag{16}$$

$$\partial_t \phi_0 + \frac{1}{2}(\vec{\nabla}\phi_0)^2 + g\eta_0 = 0 \qquad \text{at } z = \eta, \tag{17}$$

$$\partial_z \phi_0 = \partial_t \eta_0 + \vec{\nabla}_h \phi_0 \cdot \vec{\nabla}_h \eta_0 \qquad \text{at } z = \eta, \tag{18}$$

$$\partial_z \phi_0 = s(\partial_t n_0 + \vec{\nabla}_h \phi_0 \cdot \vec{\nabla}_h n_0) \qquad \text{at } z = -H, \tag{19}$$

$$\partial_t n_0 = -\omega_0 n_0 + \alpha {n_0}^2 - \beta {n_0}^3 + \delta \vec{\nabla}_h^2 n_0 \qquad \text{at } z = -H, \tag{20}$$

with $\mathscr{O}(1)$ seagrass mortality and bed shear stress

$$\omega_0 = \omega_b + \omega_c \tau_{b0}, \tag{21}$$

$$\tau_{b0} = \frac{\rho f_w \sigma}{2\pi} \int_{t-\frac{2\pi}{\sigma}}^{t} \left(\vec{\nabla}_h \phi_0\right)^2_{(x,y,-H,t')} dt'. \tag{22}$$

Similarly, the $\mathscr{O}(\varepsilon)$ equations for perturbation state $\vec{\chi}_1$ are

$$\vec{\nabla}^2 \phi_1 = 0 \qquad \text{in } \mathscr{D}, \tag{23}$$

$$\partial_t \phi_1 + \vec{\nabla}\phi_0 \cdot \vec{\nabla}\phi_1 + g\eta_1 = 0 \qquad \text{at } z = \eta, \tag{24}$$

$$\partial_z \phi_1 = \partial_t \eta_1 + \vec{\nabla}_h \eta_0 \cdot \vec{\nabla}_h \phi_1 + \vec{\nabla}_h \phi_0 \cdot \vec{\nabla}_h \eta_1 \qquad \text{at } z = \eta, \tag{25}$$

$$\partial_z \phi_1 = s(\vec{\nabla}_h \phi_0 \cdot \vec{\nabla}_h n_1 + \vec{\nabla}_h n_0 \cdot \vec{\nabla}_h \phi_1) \qquad \text{at } z = -H, \tag{26}$$

$$\partial_t n_1 = -\, \omega_c \tau_{b1} n_0 - \omega_1 n_1 + \delta \vec{\nabla}_h^2 n_1 \qquad \text{at } z = -H, \tag{27}$$

with $\mathscr{O}(\varepsilon)$ bed shear stress and seagrass mortality

$$\tau_{b1} = \frac{\rho f_w \sigma}{\pi} \int_{t-\frac{2\pi}{\sigma}}^{t} \left(\vec{\nabla}_h \phi_0 \cdot \vec{\nabla}_h \phi_1\right)_{(x,y,-H,t')} dt', \tag{28}$$

$$\omega_1 = \omega_0 - 2\alpha n_0 + 3\beta {n_0}^2. \tag{29}$$

In equation (26), the term $s\partial_t n_1$ was neglected, given that the local rate of change in topographic elevation is typically much smaller than vertical water flow induced by topographic relief, $\partial_z \phi_1$.

Basic state solution

Under specific simplifying assumptions, the $\mathscr{O}(1)$ hydrodynamic equations (16) - (19) reduce to the linearized equations for surface gravity waves, which have a well-known analytical solution[1]. Firstly, the free surface at $z = \eta$ can be replaced by a fixed surface at $z = 0$ under the assumptions that wave steepness is small and that the real water depth, $\eta + H$, is approximately equal to H, i.e.

$$a\kappa << 1 \;,\; \frac{a}{H} << 1, \tag{30}$$

with $\mathscr{O}(1)$ wave amplitude a and wavenumber κ. Secondly, requiring that equations (17) and (18) can be linearized and that $\partial_t \phi_0$ is balanced by $g\eta_0$ yields two additional requirements, i.e.

$$|\phi_0| \approx \frac{ga}{\sigma} \;,\; a\kappa << \frac{\sigma^2}{g\kappa}. \tag{31}$$

We will first assume that the latter scaling relation is valid and will confirm this afterwards. Thirdly, we assume that n_0 is a constant and will verify this afterwards. Equations (16) - (19) then can be linearized and written as

$$\begin{aligned}
\vec{\nabla}^2\phi_0 &= 0 && \text{in } \mathscr{D}_0, && (32)\\
\partial_t\phi_0 + g\eta_0 &= 0 && \text{at } z=0, && (33)\\
\partial_z\phi_0 &= \partial_t\eta_0 && \text{at } z=0, && (34)\\
\partial_z\phi_0 &= 0 && \text{at } z=-H_0, && (35)
\end{aligned}$$

where $\mathscr{D}_0$ is as $\mathscr{D}$ but with vertical range $-H_0 \leq z \leq 0$. Here, bottom boundary level H could be approximated by a fixed reference level,

$$H_0 = h - s\,n_0, \tag{36}$$

since $|n_1| << |n_0|$, $|n_2| << |n_1|$, etc., by definition of the series expansion of n.

When assuming that ϕ_0 can be written as a plane wave with a fixed vertical structure, travelling in the horizontal direction, the well-known linear gravity wave solution is found, i.e.

$$\eta_0 = a\cos(\kappa x - \sigma t), \tag{37}$$

$$\phi_0 = \frac{\sigma a}{\kappa}\frac{\cosh\left[\kappa(z+H_0)\right]}{\sinh\left[\kappa H_0\right]}\sin(\kappa x - \sigma t), \tag{38}$$

$$\sigma^2 = g\kappa\tanh\left[\kappa H_0\right]. \tag{39}$$

This is a travelling plane harmonic wave with wave amplitude a, wavenumber κ and angular frequency σ. The latter equation is the dispersion relation. Note that the wave vector was chosen to point in the x-direction, without loss of generality. The first assumption in (31) can now be verified. The second requirement of (31) becomes

$$a\kappa << \tanh(\kappa H_0), \tag{40}$$

which only poses an additional requirement with respect to (30) when $\kappa H_0 << 1$. In this latter case (the shallow water limit), $\tanh(\kappa H_0) \approx \kappa H_0$, such that $a/H_0 << 1$. In other words, the second requirement of (31) is consistent with (30).

Under the assumption that the $\mathscr{O}(1)$ seagrass density is constant in space and time, equation (20) reduces to

$$0 = -\omega_0 n_0 + \alpha {n_0}^2 - \beta {n_0}^3 \quad \text{at } z=-H_0, \tag{41}$$

and the bed shear stress becomes

$$\tau_{b0} = \frac{\rho f_w}{2}\left(\frac{\sigma a}{\sinh(\kappa H_0)}\right)^2. \tag{42}$$

Truncating $|\vec{U}_b|^2$ in (10) after $\mathscr{O}(1)$, we find

$$|\vec{U}_b| \approx \frac{\sigma a}{\sinh(\kappa H_0)}. \tag{43}$$

Wavenumber κ and seagrass density n_0 can now be solved (numerically) from equations (39) and (41), after which state variables η_0 and ϕ_0 can also be found. The solution of the basic state, for one example of parameter settings, is shown in the Results section of the main manuscript.

Perturbation equations

Whereas the linearized equations derived in the previous subsection allow for determination of the linear stability of basic state $\vec{\chi}_0$, the linearisation assumptions inevitably break down when seagrass-induced topographic modulations continue to grow in amplitude. In the full nonlinear seagrass equation (15), the competitive interaction term $-\beta n^3$ ensures saturation of seagrass growth. We anticipate that, by allowing nonlinear terms in the linearized seagrass equation (27), saturation of seagrass-induced topographic modulations will ensure that the assumptions used to linearize the hydrodynamic equations (11) - (14) remain valid. We expect that a set of fully linear perturbation-state wave equations combined with a nonlinear perturbation-state seagrass equation will lead to a physically appropriate quasi-equilibrium state, while avoiding the computational difficulties involved in numerically solving the fully nonlinear wave equations.

Thus, instead of assuming $|n_m| = \mathscr{O}(\varepsilon^m)$ and expanding

$$n = n_0 + n_1 + n_2 + ...,$$

we now define

$$n(x,y,t) = n_0 + n_1(x,y,t), \tag{44}$$

and we expect that $|n_1| \lesssim |n_0|$ due to the growth saturation term that will be introduced. Note that we have now directly assumed that n_0 is constant, as was found in the previous subsection.

With this notation, the basic- and perturbation-state approximations of bottom boundary equation (14) become

$$\partial_z \phi_0 = s\vec{\nabla}_h \phi_0 \cdot \vec{\nabla}_h n_1,$$
$$\partial_z \phi_1 = s\vec{\nabla}_h \phi_1 \cdot \vec{\nabla}_h n_1.$$

The term on the right-hand side of the basic-state equation can be neglected (consistent with equation 35), given that $|s\vec{\nabla}_h \phi_0 \cdot \vec{\nabla}_h n_1|/|\partial_z \phi_0| \approx \kappa |s\, n_1|$, which is much smaller than 1 since seagrass-induced topographic modulations have a typical amplitude $|s\, n_1|$ of about 1m,[10]. However, the term $s\vec{\nabla}_h \phi_0 \cdot \vec{\nabla}_h n_1$ does balance with $\partial_z \phi_1$ in the perturbation equation, where it dominates over the term $s\vec{\nabla}_h \phi_1 \cdot \vec{\nabla}_h n_1$. In conclusion, we replace the fully linear perturbation equations (23 - 27) by the following final perturbation equations, with linearized hydrodynamics and nonlinear seagrass dynamics:

$$\vec{\nabla}^2 \phi_1 = 0 \qquad \text{in } \mathscr{D}_0, \tag{45}$$

$$\partial_t \phi_1 + \vec{\nabla}\phi_0 \cdot \vec{\nabla}\phi_1 + g\eta_1 = 0 \qquad \text{at } z = 0, \tag{46}$$

$$\partial_z \phi_1 = \partial_t \eta_1 + \vec{\nabla}_h \eta_0 \cdot \vec{\nabla}_h \phi_1 + \vec{\nabla}_h \phi_0 \cdot \vec{\nabla}_h \eta_1 \qquad \text{at } z = 0, \tag{47}$$

$$\partial_z \phi_1 = s\vec{\nabla}_h \phi_0 \cdot \vec{\nabla}_h n_1 \qquad \text{at } z = -H_0, \tag{48}$$

$$\partial_t n_1 = -\,\omega_c \tau_{b1}\,(n_0 + n_1) - \omega_1 n_1 + \alpha_1 {n_1}^2 - \beta {n_1}^3 + \delta \vec{\nabla}_h^2 n_1 \qquad \text{at } z = -H_0, \tag{49}$$

with τ_{b1} as in (28), ω_1 as in (29), and the perturbation-state facilitative interaction coefficient α_1 defined as

$$\alpha_1 = \alpha - 3\beta n_0. \tag{50}$$

Note that the fact that $|\eta_1| << |\eta_0|$ and $|n_1| \lesssim |n_0|$ justified approximating the water surface level $z = \eta$ by a fixed level $z = 0$ and replacing the bottom boundary level $z = -H$ by a constant level $z = -H_0$ in these perturbation equations.

Linear stability of the non-vegetated state

Equation (41) has two physically relevant solutions, namely a non-vegetated state ($n_0 = 0$) and a vegetated state ($n_0 > 0$). As explained in the main manuscript, the linear stability analysis of the vegetated state is not trivial and is therefore inferred from numerical time-integration of perturbation equations (45 - 49). These numerical analyses will be discussed in the next section. Linear stability of the non-vegetated state, however, can be calculated analytically, as will be shown here.

Consider a small, uniform perturbation ν to any of the uniform equilibrium solutions n_0 of equation (41). The evolution of this uniform perturbation can be written as

$$\partial_t \nu = \partial_t (n_0 + \nu) - \partial_t n_0. \tag{51}$$

Using equations (41) and (42), we can write

$$\partial_t n_0 = -\omega_0 n_0 + \alpha {n_0}^2 - \beta {n_0}^3 \quad \text{at } z = -H_0, \tag{52}$$

with

$$\omega_0 = \omega_b + \frac{\omega_c \rho f_w}{2} \left(\frac{\sigma a}{\sinh[\kappa(h - s\, n_0)]} \right)^2. \tag{53}$$

When we assume that perturbing n_0 with ν does not lead to significant changes in κ, equation (51) can be written as

$$\partial_t \nu = -\left[\omega_b + \frac{\omega_c \rho f_w}{2} \left(\frac{\sigma a}{\sinh[\kappa(h - s\,(n_0 + \nu))]} \right)^2 \right] (n_0 + \nu) + \alpha (n_0 + \nu)^2 - \beta (n_0 + \nu)^3 \tag{54}$$
$$+ \left[\omega_b + \frac{\omega_c \rho f_w}{2} \left(\frac{\sigma a}{\sinh[\kappa(h - s\, n_0)]} \right)^2 \right] n_0 - \alpha {n_0}^2 + \beta {n_0}^3.$$

Expanding this expression around $\nu = 0$ and truncating after first order yields the linear perturbation equation, i.e.

$$\partial_t \nu = -\left[\omega_b + \frac{\omega_c \rho f_w}{2}\left(\frac{\sigma a}{\sinh(\kappa h)}\right)^2\right]\nu = \lambda_\nu\ \nu. \tag{55}$$

We define the critical wave amplitude a^* as the value of a where the eigenvalue λ_ν becomes zero, i.e.

$$a^*_\pm = \pm\, i\sqrt{\frac{2\omega_b}{\omega_c \rho f_w}}\ \frac{\sinh(\kappa h)}{\sigma}. \tag{56}$$

For the current choice of real-valued parameters and $\omega_b < 0$ (see Supplementary Table S1), the critical wave amplitude is a^*_+ and λ_ν is positive for $a < a^*_+$ and negative for $a > a^*_+$. That is, the non-vegetated solution ($n_0 = 0$) is linearly unstable when wave forcing amplitude is weaker than a^*_+ and linearly stable when wave forcing is stronger than a^*_+.

Numerical solutions

In this section, we describe the methods used to numerically solve the uniform basic state $\vec{\chi}_0$ and to numerically time-integrate the equations for perturbation state $\vec{\chi}_1$.

Numerical solution of the uniform basic state

To solve the uniform basic state, dispersion relation (39) and seagrass equation (41) need to be solved numerically for water wavenumber κ and seagrass density n_0. Surface elevation η_0 and velocity potential ϕ_0 then follow from equations (37) and (38).

In this study we consider monochromatic waves, hence angular wave frequency σ is a constant. To fix σ, we solve dispersion relation (39) analytically by first assuming an absence of seagrass (i.e., $H_0 = h$) and choosing $\tilde{\kappa}_n/2$ as estimate for κ, where $\tilde{\kappa}_n$ is the average seagrass pattern wavenumber observed around Mallorca[11]. Here, we have a priori hypothesized that the seagrass bedforms are formed by Bragg resonance (the hypothesis which is then tested in this study), such that the incoming water wavenumber is half the seagrass pattern wavenumber[9].

We then numerically solve the uniform basic state as a function of wave amplitude a. Starting at $a = 0$, seagrass equation (41) can be solved analytically for n_0. This value is then used to solve dispersion equation (39) numerically, using $\tilde{\kappa}_n/2$ as initial guess for κ. Then, wave amplitude a is increased in small steps. For each increment, the seagrass equation and dispersion relation are solved together, using the solution of n_0 and κ at the previous value of a as initial guess for the numerical solver. This procedure is used both for the unvegetated solution ($n_0 = 0$) and the vegetated solution ($n_0 > 0$). Parameters used to solve $\vec{\chi}_0$ are listed in Supplementary Table S1, where $[n]$ denotes the unit of seagrass density n (which, in our study, we assume to be dimensionless).

We obtained the values of the seagrass-related parameters (δ, ω_b, ω_c, α and β) as follows. First, we started from the seagrass equation described in previous studies[5,11], and simplified this equation by retaining only the terms responsible for facilitative and competitive interaction, local mortality and lateral diffusive dispersion, since we hypothesize that these terms alone (together with the effect of wave-induced bed shear stress on seagrass mortality) are sufficient to explain seagrass patterning. Second, we slightly adjusted the values of α and β, such that seagrass density has only one solution for each value of control parameter ω. This is done to avoid bistability, as was present in the original model[5] but which would distract the attention of our study from its focus on pattern formation. Third, we made the mortality rate ω explicitly dependent on the wave-induced bed shear stress, via equation (8). Herein, we chose a negative value for the background mortality, ω_b, such that a vegetated meadow exists in the absence of wave forcing. The coupling strength ω_c between bed shear stress and seagrass mortality was then chosen such that, for a realistic range of water wave amplitudes, the seagrass density gradually decreases and eventually becomes zero. Fourth, lateral seagrass dispersion coefficient δ was chosen such that the diffusion length scale, $2\sqrt{\delta/\Delta t}$, is of the order of the horizontal grid cell spacing Δx, to avoid numerical instabilities. Finally, we assigned the units of seconds and meters to the independent variables time and space, respectively, in the seagrass equation, which was dimensionless in its original form[5]. Given this same time- and spatial scale, the seagrass dynamics can be directly coupled to the hydrodynamics. Although the current study is theoretical and aims purely at testing the hypothesis that wave reflection can explain seagrass self-organization, it is desirable to further calibrate the seagrass-related model parameters based on field or literature study in follow-up research.

Note that due to the modifications described above, the values of seagrass-related parameters (δ, ω_b, ω_c, etc.) are not directly comparable to the values reported in previous studies[5,11]. This is because, in our model, we need to bridge the gap between the hydrodynamic timescales (wave dynamics, on the order of seconds) and the biogeomorphic timescales (seagrass dynamics, on the order of months to years). We bridge this timescale difference by effectively speeding up the simulated (bio)geomorphic evolution. This approach is applied often in morphological modelling and consists in multiplying the (bio)geomorphic dynamics

with a so-called morphological acceleration factor to avoid excessively long computation times[12]. In our study, we chose to write the seagrass equation (15) in the same time-coordinate t as the hydrodynamic equations (11-14), and to speed up the seagrass dynamics relative to the wave dynamics by multiplying the biogeomorphic parameters with a large morphological acceleration factor. For this reason, the rates of seagrass dynamics are much larger in our model than in other studies[5,11].

Supplementary Table S1. Physical model parameter values. This is the default set of parameters used in all figures shown in the main manuscript and Supplementary Information. Only in Supplementary Figure S4, the values of ω_c is varied (as specified in that figure).

Par.	Value	Unit	Meaning	Ref.
a	0.0 - 3.0	m	Incoming wave amplitude	-
g	9.81	m s^{-2}	Gravitational acceleration coefficient	-
h	40	m	Water depth in absence of seagrass	11
λ_n	62.5	m	Observed average seagrass pattern wavelength	11
$\tilde{\kappa}_n$	$2\pi/\lambda_n$	m^{-1}	Observed average seagrass pattern wavenumber	11
ρ	1025	kg m^{-3}	Sea water density	-
f_w	0.01	-	Wave friction factor	13
δ	16	m^2 s^{-1}	Lateral seagrass dispersion coefficient	-
ω_b	-2	s^{-1}	Constant background value of the net seagrass mortality rate	-
ω_c	4	N^{-1} m^2 s^{-1}	Coupling strength between bed shear stress and seagrass mortality	-
α	0.75	$[n]^{-1}$ s^{-1}	Facilitative seagrass interaction coefficient	-
β	0.50	$[n]^{-2}$ s^{-1}	Competitive seagrass interaction coefficient	-
s	12	m $[n]^{-1}$	Topography coefficient, translating seagrass density to topographic elevation	-

Numerical solution of the perturbation state

Starting from the (vegetated) basic state solution $\vec{\chi}_0$, which was computed following the approach in the previous subsection, the perturbation equation equations (45) - (49) are numerically integrated over time. Physical model parameters are given in Supplementary Table S1. Non-physical parameters, related to the numerical method used, are listed in Supplementary Table S2.

Since the basic-state wave field is a plane wave travelling in the x-direction, we can ignore any variations in the y-direction. The perturbation equations are solved numerically on an equidistant grid in (x,z)-space. The length of this domain (along the x-direction, i.e. from left to right) is such that it fits exactly N_{waves} water waves of wavelength $\lambda = 2\pi/\kappa$. Vertically, the domain ranges from $z = -H_0$ to $z = 0$. Aiming for a horizontal grid cell spacing of Δx_{aim}, the number of grid cells N_x is computed by dividing the horizontal extent of the domain by Δx_{aim} and then rounding to the nearest odd integer. The exact horizontal grid spacing Δx then becomes the domain length $(N_{waves}\lambda)$ divided by N_x. The same approach is followed in the vertical direction, where Δ_z is the vertical grid spacing that is aimed for. The number of vertical grid cells N_z is then the nearest odd integer number of grid cells with this approximate grid spacing that fits in the vertical extent between $z = 0$ and $z = -H_0$. Since H_0 is a function of n_0 (and hence of wave amplitude a), the number of grid cells N_z varies dependent on wave amplitude a.

Since the basic state $(\phi_0(x,z,t),\ \eta_0(x,t),\ n_0)$ is known and decoupled from the perturbation state, the basic state can simply be imposed for each grid cell and during each timestep of the numerical time-integration of the equations for the perturbation state $(\phi_1(x,z,t),\ \eta_1(x,t),\ n_1(x,t))$. Initially, there is no perturbation, i.e. $\eta_1 = 0$ and ϕ_1 is constant (here chosen to be 1). To trigger the modulation instability, uniformly distributed random numbers within the range $[-A_{n1}, A_{n1}]$ are taken as initial condition $n_1(x,t=0)$. For cells for which total seagrass density $(n_0 + n_1)$ would be negative, n_1 is adjusted such that $n_0 + n_1 = 0$ there. Flow velocities are calculated from ϕ_1 (i.e., $u_1 = \partial_x \phi_1$ and $w_1 = \partial_z \phi_1$) using the standard finite-difference operators (central difference for grid cells in the interior, forward or backward difference for boundary cells) with $\mathcal{O}(\Delta x^2)$ and $\mathcal{O}(\Delta z^2)$ accuracy[14]. Since calculation of τ_{b1} requires the values of the integrand of (28) for all timesteps of the preceding wave period, the initial condition of τ_{b1} is calculated by assuming that the integrand has remained constant throughout the entire preceding wave period. After this initial time step, the perturbation equations are time-integrated numerically. A time step size Δt is chosen, such that exactly $N_{\Delta t}$ timesteps fit in one wave period T. The time-integration procedure is as follows.

Firstly, the kinematic boundary equation at the water surface (47) is applied. To allow time integration of this equation, it is first rewritten as

$$\partial_t \eta_1 = F_{\eta 1}(x,t) \qquad \text{at } z = 0, \tag{57}$$

with $F_{\eta 1}$ a function representing the remaining terms in equation (47). Standard Euler forward time integration would yield η_1,

i.e.

$$\eta_1(x,t+\Delta t) = \eta_1(x,t) + \Delta t \cdot F_{\eta 1}(x,t) \quad \text{at } z=0.$$

However, to minimize reflection of the perturbation wave (ϕ_1, η_1) on the lateral domain boundaries, a so-called sponge layer needs to be implemented first[15–17]. A sponge layer is implemented on both lateral ends of the domain to mimic an infinitely long domain. Within this sponge layer, a damping term (f_{SL} and f_{SR} for the sponge layers on the left- and right-hand side, respectively) is imposed whose strength increases gradually from the interior of the domain towards the boundary, i.e.

$$\eta_1(x,t+\Delta t) = \eta_1(x,t) + \Delta t \cdot [F_{\eta 1}(x,t) + f_{SL}(x)(\eta_{1L} - \eta_1(x,t)) + f_{SR}(x)(\eta_{1R} - \eta_1(x,t))] \quad \text{at } z=0, \tag{58}$$

with damping terms

$$f_{SL}(\xi_L) = A_S\, {\xi_L}^{P_S} \text{ and } f_{SR}(\xi_R) = A_S\, {\xi_R}^{P_S}, \tag{59}$$

where A_S is the amplitude of the damping functions and ξ_L is the horizontal coordinate that linearly increases from 0 to 1 within the left sponge layer (i.e., increases in the negative x-direction) and ξ_R linearly increases from 0 to 1 in the right sponge layer (i.e., increases in the positive x-direction), see Supplementary Figure S2d. Outside of the sponge layers, f_{SL} and f_{SR} are zero. P_S is the power of the damping function, i.e. it determines how quickly the damping term increases within the sponge layer. Furthermore, η_{1L} and η_{1R} are the values of η_1 that are imposed at the outermost left and right boundary. We choose both values to be zero, such that the perturbation wave field is completely damped out at the end of the sponge layers and wave reflection is minimized. Here, both sponge layers are chosen to fit exactly N_S water wavelengths λ.

Secondly, similarly to the kinematic surface boundary condition, the dynamic surface boundary condition, equation (46), is rewritten as

$$\partial_t \phi_1 = F_{\phi 1}(x, z=0, t) \quad \text{at } z=0, \tag{60}$$

and the same damping functions f_{SL} and f_{SR} are applied to dampen $\phi_1(x,z=0,t)$ towards a constant value of $\phi_{1L} = 1$ and $\phi_{1R} = 1$ at the outermost left and right boundaries, i.e.

$$\phi_1(x,z,t+\Delta t) = \phi_1(x,z,t) + \Delta t \cdot \left[F_{\phi 1}(x,z,t) + f_{SL}(x)(\phi_{1L} - \phi_1(x,z,t)) + f_{SR}(x)(\phi_{1R} - \phi_1(x,z,t))\right] \quad \text{at } z=0. \tag{61}$$

The constant values ϕ_{1L} and ϕ_{1R} are imposed as boundary conditions on the outermost left and right boundaries, for all values of z.

Thirdly, the Laplace equation (45) is solved numerically for ϕ_1. An iterative Laplacian inversion technique (Gauss-Seidel method with successive over-relaxation) is used[14], where r is the residual used to let the iterative process converge and the relaxation parameter p_r determines the speed of convergence. Laplacian inversion is performed for each time step. Initially, the solution of ϕ_1 from the previous time step is used as the first guess in the iterative inversion method. Then, the boundary conditions at the left and right boundaries (ϕ_{1L} and ϕ_{1R}) are imposed as well as the surface boundary condition computed from the dynamic surface boundary condition (as discussed above). Then, one Laplacian inversion iteration is performed, only for the interior domain (i.e., excluding the left, right, surface and bottom boundaries). After that, the bottom boundary condition, equation (48), is discretized to obtain the bottom boundary condition for ϕ_1. This boundary condition is then imposed as well. After this entire procedure, the relative error at iteration step $k+1$ is calculated, i.e.

$$e^{k+1} = \left| \frac{{\phi_1}^{k+1} - {\phi_1}^{k}}{{\phi_1}^{k+1}} \right|, \tag{62}$$

where ${\phi_1}^{k+1}$ is the solution of the Laplacian inversion method after iteration $k+1$. This solution then becomes the initial guess of ϕ_1 for the next iteration, and so on. This iteration loop is repeated until the relative error becomes smaller than a critical value e_{crit}, i.e. until the solution has converged.

Fourthly, once the Laplacian inversion process has converged, the basic-state wave field (ϕ_0, η_0) is updated (i.e., computed for this new time step, $t+\Delta t$). Using these values and the updated values for ϕ_1 and η_1, we calculate the instantaneous perturbation bed shear stress during this time step, which is proportional to the integrand in equation (28).

Fifthly, this entire process (i.e., numerically solving the perturbation wave field and calculating the basic-state wave field for a new time step) is repeated $N_{\Delta t}$ times, i.e. for one entire wave period T. Hence, we now know the instantaneous perturbation bed shear stress values for all time steps within this entire wave period. Following equation (28), the perturbation bed shear stress averaged over this wave-period, τ_{b1}, can be computed.

Sixthly, the perturbation seagrass field is updated, i.e.

$$n_1(x,t+T) = n_1(x,t) + \Delta t \cdot \partial_t n_1(x,t) \qquad \text{at } z = -H_0, \tag{63}$$

where $\partial_t n_1(x,t)$ is given by the seagrass equation (49). Note that $\partial_t n_1(x,t)$ is multiplied by time step Δt to obtain the difference Δn_1, but that this difference is used to compute n_1 at the next *wave period* $(t+T)$ rather than the next time step $(t+\Delta t)$. This is because there is a lag effect, i.e. the development of n_1 depends on the aggregated bed shear stress over the entire preceding wave period. Lateral boundary conditions for n_1 are given by

$$\partial_x n_1 = 0 \qquad \text{at } x = \pm L. \tag{64}$$

The numerical time-integration of ϕ_1 and η_1 (every time step Δt) and n_1 (every wave period T) is continued for a certain number of wave periods. Here, we only consider the initial phase of this time-integration, i.e. the phase during which the linearization conditions are (reasonably) satisfied. The time evolution beyond this, when non-linear effects start to become important, is left for future studies. All gradient operators are discretized with the standard finite-difference schemes (central difference for grid cells in the interior, forward or backward difference for boundary cells) with $\mathcal{O}(\Delta x^2)$ or $\mathcal{O}(\Delta z^2)$ accuracy[14].

Supplementary Table S2. Numerical model settings.

Par.	Value	Unit	Meaning
Δx_{aim}	2.5	m	Horizontal grid cell spacing that is aimed for
Δz_{aim}	2.5	m	Vertical grid cell spacing that is aimed for
N_{waves}	20	-	Number of water wavelengths that fits in the model domain
N_S	3	-	Number of water wavelengths that fits in either sponge layer
A_S	8	s^{-1}	Amplitude of the damping function in the sponge layers
P_S	3	-	Power of the damping function in the sponge layers
A_{n1}	$5*10^{-3}$	$[n]$	Amplitude of the uniformly distributed random numbers (initial condition for n_1)
$N_{\Delta t}$	2^9	-	Number of time steps Δt per wave period T
p_r	1.5	-	Relaxation parameter used for Laplacian inversion
e_{crit}	$1*10^{-5}$	-	Relative error used as criterion to decide if Laplacian inversion has converged yet

Power spectra and linear growth rates

To estimate linear growth/decay rates of the modulation n_1, the time-development of seagrass density perturbation $n_1(x,t)$ is analyzed. First, the power spectrum of n_1, as a function of wavenumber κ_{n1} is calculated for each wave period T. More specifically, we calculate the single-sided amplitude spectrum as a function of wavenumber and time, $P_{n1}(\kappa_{n1},T)$. First, we calculate the Fast Fourier Transform of the seagrass perturbation field n_1 within the interior part of the model domain, i.e. excluding the sponge layers. With this, the two-sided spectrum and from that the single-sided spectrum are calculated.

Dimensionless linear growth rates $r_{n1}(\kappa_{n1})$ are given by

$$P_{n1}(\kappa_{n1},t) = P_{n1}(\kappa_{n1},0)\ \exp\left[r_{n1}(\kappa_{n1})\ \frac{t}{T}\right]. \tag{65}$$

As an illustration, Supplementary Figure S3 shows the time-evolution of $P_{n1}(\kappa_{n1} = 2\kappa, t)$ (normalized by its initial value and shown on logarithmic scale), for different wave forcing strengths. Seagrass modulations with wavenumber 2κ show the fastest growth rate, and we thus indicate this dominant pattern wavenumber as $\kappa_{n1}{}^*$.

To estimate $r_{n1}(\kappa_{n1})$ from the numerical simulations, we calculate $P_{n1}(\kappa_{n1},T)$ for each simulated time step T. We then invert (65) and approximate $r_{n1}(\kappa_{n1})$ by considering the relative growth or decay over an interval of N wave periods, i.e.

$$r_{n1}(\kappa_{n1}) \approx \frac{1}{(N-1)} \ln\left[P_{n1}(\kappa_{n1},NT)/P_{n1}(\kappa_{n1},T)\right]. \tag{66}$$

Determination of the modulation instability

The location of the modulation instability, a_{MI} is estimated from the numerical time-integration of n_1. Since the calculations (66) of $r_{n1}(\kappa_{n1})$ reveal that modulations with $\kappa_{n1} = 2\kappa$ grow the fastest (see the main manuscript), we use the relative growth of modulations at this wavenumber to determine the location of the modulation instability. The value of a where the relative growth goes from negative (decay; for $a < a_{MI}$) to positive (growth; for $a > a_{MI}$ is identified as the modulation instability. The modulation instability is derived from numerical simulations, i.e. from trajectories like those shown in Supplementary Figure S3.

Supplementary Figure S4 shows how the location of the modulation instability changes with changing ω_c.

References

1. Holthuijsen, L. H. Waves in oceanic and coastal waters (Cambridge university press, 2010).
2. Vacchi, M. et al. Biogeomorphology of the mediterranean posidonia oceanica seagrass meadows. Earth Surf. Process. Landforms **42**, 42–54 (2017).
3. Lo Iacono, C. et al. Very high-resolution seismo-acoustic imaging of seagrass meadows (mediterranean sea): Implications for carbon sink estimates. Geophys. Res. Lett. **35** (2008).
4. Serrano, O., Mateo, M., Renom, P. & Julià, R. Characterization of soils beneath a posidonia oceanica meadow. Geoderma **185**, 26–36 (2012).
5. Ruiz-Reynés, D., Schönsberg, F., Hernández-García, E. & Gomila, D. General model for vegetation patterns including rhizome growth. Phys. Rev. Res. **2**, 023402 (2020).
6. Swart, D. Offshore sediment transport and equilibrium beach profiles. PhD Diss. Dep. Civ. Eng. Delft Univ. Technol. (1974).
7. van der Heide, T. et al. Positive feedbacks in seagrass ecosystems: implications for success in conservation and restoration. Ecosystems **10**, 1311–1322 (2007).
8. Aagaard, T., Christensen, D. F. & Hughes, M. G. Field measurements of shear stress and friction in the surf zone. Earth Surf. Process. Landforms **46**, 385–398 (2021).
9. Davies, A. & Heathershaw, A. Surface-wave propagation over sinusoidally varying topography. J. Fluid Mech. **144**, 419–443 (1984).
10. Kendrick, G. A., Marbà, N. & Duarte, C. M. Modelling formation of complex topography by the seagrass posidonia oceanica. Estuarine, Coast. Shelf Sci. **65**, 717–725 (2005).
11. Ruiz-Reynés, D. et al. Fairy circle landscapes under the sea. Sci. Adv. **3**, e1603262 (2017).
12. Morgan, J. A. et al. The use of a morphological acceleration factor in the simulation of large-scale fluvial morphodynamics. Geomorphology **356**, 107088 (2020).
13. Newgard, J. P. & Hay, A. E. Turbulence intensity in the wave boundary layer and bottom friction under (mainly) flat bed conditions. J. Geophys. Res. Ocean. **112** (2007).
14. Cushman-Roisin, B. & Beckers, J.-M. Introduction to geophysical fluid dynamics: physical and numerical aspects (Academic press, 2011).
15. Clément, A. Coupling of two absorbing boundary conditions for 2d time-domain simulations of free surface gravity waves. J. Comput. Phys. **126**, 139–151 (1996).
16. Papoutsellis, C. E. Numerical simulation of non-linear water waves over variable bathymetry. Procedia Comput. Sci. **66**, 174–183 (2015).
17. Carmigniani, R. A. & Violeau, D. Optimal sponge layer for water waves numerical models. Ocean. Eng. **163**, 169–182 (2018).

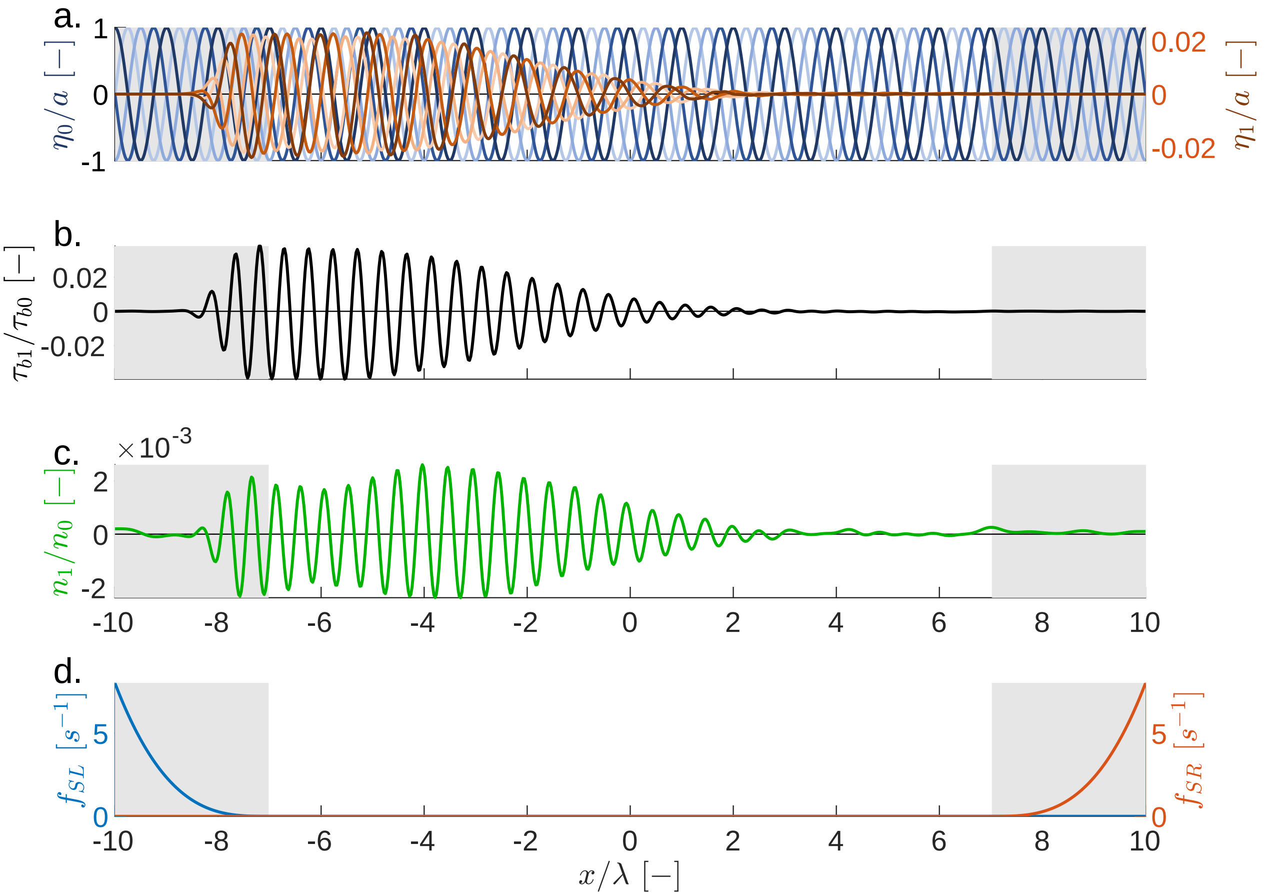

Supplementary Figure S2. Simulation results of the same simulation shown in Figure 3 of the main manuscript, but now shown for the full simulation domain (including sponge layers). **a**. Incoming wave field (i.e., basic-state water surface elevation η_0) is shown in blue (darkness increasing with time, i.e. waves travel to the right) and reflected wave field (i.e., perturbation-state surface elevation η_1) is shown in orange shades (darkness increasing with time, i.e. waves travel to the left). Water surface elevations are normalized by forcing wave amplitude a and are shown at four instances within the 25th wave period ($t/T = 24\frac{1}{4}$, $24\frac{2}{4}$, $24\frac{3}{4}$ and 25), with T the wave period. The full horizontal domain is shown here; sponge layers span a width of 3λ on the left and right border and are indicated with gray shading. **b** Perturbation-state bed shear stress τ_{b1} (normalized by basic-state bed shear stress τ_{b0}), at $t/T = 25$. **c** Perturbation-state seagrass density n_1 (normalized by basic-state seagrass density n_0), at $t/T = 25$. Horizontal direction x is normalized by the incoming water wavelength $\lambda = 2\pi/\kappa$. **d** The damping functions f_{SL} and f_{SR} in the sponge layers.

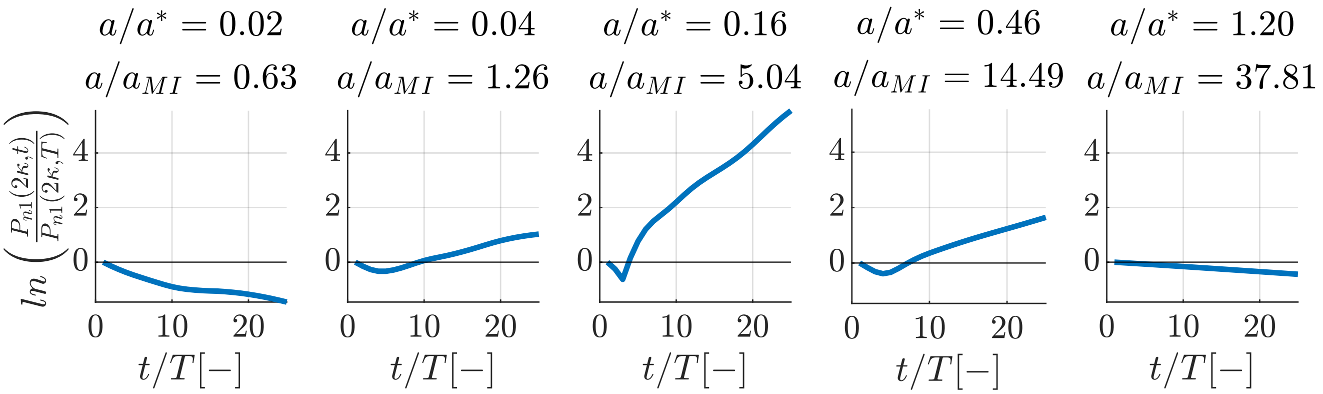

Supplementary Figure S3. Spectral power of n_1 at wavenumber $\kappa_{n1} = \kappa_{n1}{}^* = 2\kappa$ as a function of time, for different wave forcing strengths. Spectral power is normalized by its initial value and logtransformed. The slope of the graphs equals the linear growth rates $r_{n1}(2\kappa)$ shown in Fig. 2c of the main manuscript. Figure titles indicate wave forcing strength, relative to the transcritical bifurcation point a^* and to the modulation instability a_{MI}.

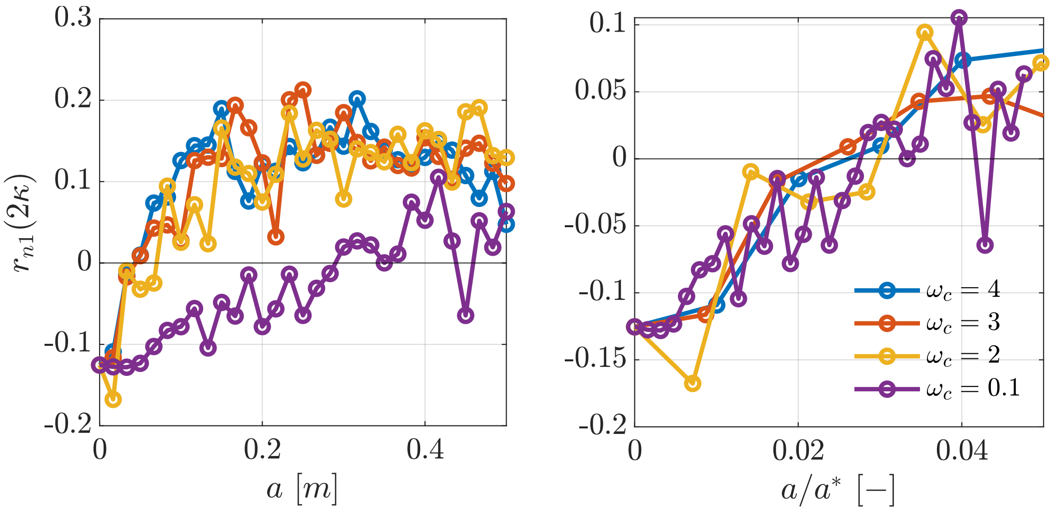

Supplementary Figure S4. Linear growth rates $r_{n1}(2\kappa)$, as in equation (66), for $N = 25$ and for different parameter values of ω_c. The intersect of the growth curves with the zero axis identifies the modulation instability, $a = a_{MI}$. Left and right panels show growth curves as a function of a and as function of a/a^*, respectively. The parameter values used are as described in Supplementary Table S1, except ω_c, which is varied (as specified in the legend).
